# Sb-Doped Metal Halide Nanocrystals: A 0D versus 3D Comparison

**DOI:** 10.1021/acsenergylett.1c00789

**Published:** 2021-05-27

**Authors:** Dongxu Zhu, Matteo L. Zaffalon, Juliette Zito, Francesca Cova, Francesco Meinardi, Luca De Trizio, Ivan Infante, Sergio Brovelli, Liberato Manna

**Affiliations:** † Nanochemistry Department Istituto Italiano di Tecnologia, 16163 Genova, Italy; ‡ Dipartimento di Scienza dei Materiali, Università degli Studi di Milano Bicocca, 20125 Milano, Italy; § Dipartimento di Chimica e Chimica Industriale, Università degli Studi di Genova, 16146 Genova, Italy

## Abstract

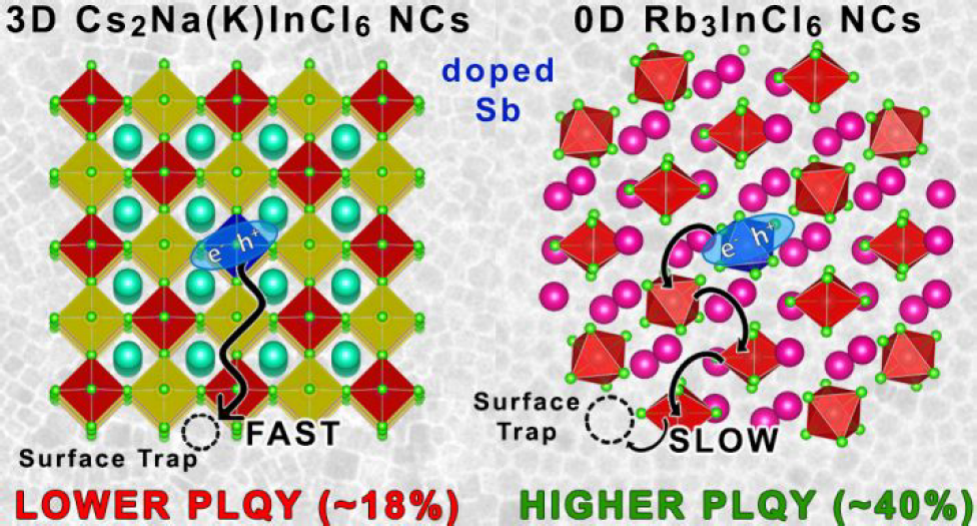

We synthesize colloidal nanocrystals (NCs) of Rb_3_InCl_6_, composed of isolated metal halide octahedra (“0D”), and of Cs_2_NaInCl_6_ and Cs_2_KInCl_6_ double perovskites, where all octahedra share corners and are interconnected (“3D”), with the aim to elucidate and compare their optical features once doped with Sb^3+^ ions. Our optical and computational analyses evidence that the photoluminescence quantum yield (PLQY) of all these systems is consistently lower than that of the corresponding bulk materials due to the presence of deep surface traps from under-coordinated halide ions. Also, Sb-doped “0D” Rb_3_InCl_6_ NCs exhibit a higher PLQY than Sb-doped “3D” Cs_2_NaInCl_6_ and Cs_2_KInCl_6_ NCs, most likely because excitons responsible for the PL emission migrate to the surface faster in 3D NCs than in 0D NCs. We also observe that all these systems feature a large Stokes shift (varying from system to system), a feature that should be of interest for applications in photon management and scintillation technologies. Scintillation properties are evaluated via radioluminescence experiments, and re-absorption-free waveguiding performance in large-area plastic scintillators is assessed using Monte Carlo ray-tracing simulations.

Lead halide perovskite nanocrystals (NCs) feature a bright and narrow photoluminescence (PL) emission that can be varied over the whole visible spectrum by simple compositional tuning.^1−5^ On the other hand, these materials are inherently toxic, due to the presence of Pb, and unstable, especially if exposed to heat, air, and moisture.^6^ Hence, the current quest is to replace such Pb-based perovskites with alternative non-toxic metal halide NCs that could exhibit similar optical properties and, ideally, higher stability.^7^ In this context, the broad family of double perovskites (DPs), having chemical formula A_2_B^+^B^3+^X_6_ and a crystal structure composed of BX_6_ corner-sharing octahedra surrounded by A^+^ cations (Scheme 1, left side), is particularly promising and offers a fertile ground for new discoveries.^8−13^ Various DP materials of the Cs_2_B^+^B^3+^Cl_6_ type (where B^+^ = Ag^+^, Na^+^ and B^3+^ = In^3+^, Bi^3+^, Sb^3+^) have been investigated in a short time span,^9,14−21^ and all of them were found to have a weak PL emission in both bulk and nanoscale dimensions, stemming from either an indirect bandgap or, in the case of direct bandgap materials, a parity-forbidden transition.^13,22−28^ Thus, in order to improve their optical properties, several strategies, including doping, alloying, or both, have been devised to improve their PL efficiency.^4,18,21,27,29−42^

**Scheme 1:**
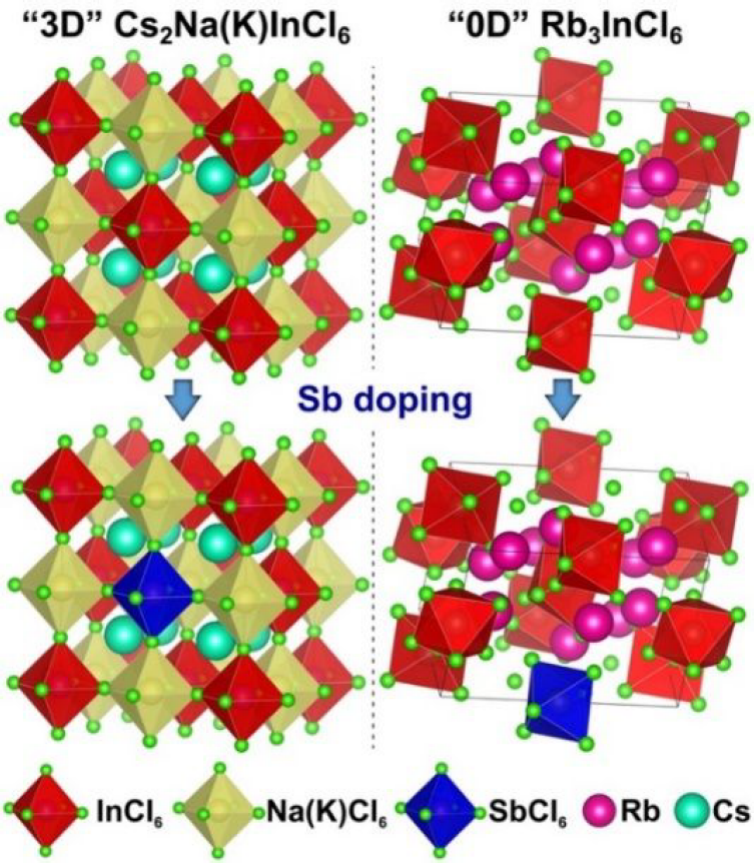
Sketch of the Crystal Lattices of Undoped and Sb-Doped “3D” Double Perovskites and “0D” Nanocrystals Studied in This Work

Among the different dopants, Sb^3+^ cations are particularly interesting, as they confer highly efficient optical emission properties to bulk DPs.^43−49^ For example, several works have demonstrated that Sb-doping of Cs_2_NaInCl_6_ DP bulk crystals yields a bright, broad (full width at half-maximum of 80 nm) emission centered at ∼450 nm with a PL quantum yield (PLQY) of ∼80%.^43,45,48^ Similarly, Noculak et al. synthesized both Cs_2_NaIn_1–*x*_Sb_*x*_Cl_6_ and Cs_2_KIn_1–*x*_Sb_*x*_Cl_6_ powders, exhibiting blue and green emission, respectively, with PLQY as high as ∼90%.^46^

Sb^3+^ cations were found to be efficient dopants also for other metal halides, such as the so-called “0D” structures: here the metal halide octahedra are isolated from each other (Scheme 1, right side).^50−55^ In this regard, various non-luminescent or weakly luminescent 0D host bulk materials (Cs_2_SnCl_6_, Rb_3_InCl_6_, Cs_3_InCl_6_, and their hydrated counterparts) were reported to show bright PL emission, with PLQY values typically reaching ∼90% when doped with Sb^3+^ cations.^51−54,56^ As a general trend, Sb-doped DPs and 0D materials are all characterized by identical near-UV absorption features that have been attributed to electronic transitions within the [SbCl_6_] octahedra, which are considered to act as sensitizers in a host matrix that is transparent to light at the band edges (due to parity-forbidden transitions).^43−47,51−55^ The absorption from [SbCl_6_] octahedra resembles an atomic-like excitation influenced by spin–orbit coupling, featuring a peak at high energies (∼280 nm, ascribed to the ^1^S_0_→^3^P_2_ transition) and a doublet at lower energies (in the 300–400 nm range, corresponding to the parity-allowed, spin-forbidden ^1^S_0_→^3^P_1_ and ^1^S_0_→^3^P_0_ transitions).^43−47,51−55^ In emission, the [SbCl_6_] octahedra act also as recombination centers by undergoing a large structural reorganization, as evidenced by the substantial Stokes shift. The shift varies from system to system: it is, for example, ∼0.91 eV in Sb-doped Cs_2_NaInCl_6_, 2.1 eV in Sb-doped Rb_2_InCl_5_·H_2_O, 1.29 eV in Sb-doped Rb_3_InCl_6_, and 1.19 eV in Sb-doped Cs_2_KInCl_6_.^46^ This variability has been attributed to the different coordination environments probed by the Sb^3+^ cations, namely [SbCl_6_] or [SbCl_5_(H_2_O)] octahedra.^52^

In this work, we aim to expand our understanding of the emission characteristics of Sb-doped metal halide systems in the NC form. In particular, we want to elucidate whether the extent of the connectivity of metal halide octahedra has any substantial influence on the optical properties of Sb-doped materials at the nanoscale. We synthesized both undoped and Sb-doped NCs of Cs_2_NaInCl_6_, Cs_2_KInCl_6_ (two different DP materials) and Rb_3_InCl_6_ (a 0D system) and carried out optical and computational analyses on them. In absorption, both 3D and 0D systems behave very similarly, while most differences lie in their emission features. The Sb-doped “0D” Rb_3_InCl_6_ NCs tend to have a markedly higher PLQY (∼40%) than the corresponding Sb-doped “3D” DP (Cs_2_NaInCl_6_ and Cs_2_KInCl_6_) NCs (∼15–18%), although both present values are well below those of the bulk crystal counterparts. Based on our computational analysis we suggest that, in the 0D case, the isolated [SbCl_6_] octahedra form self-trapped excitons (STEs) that are less likely to migrate to a defect-rich surface of the NCs, dominated by under-coordinated halide ions (which we prove to be the main source of traps). This behavior contrasts with the DP case, where [SbCl_6_] octahedra are interconnected with [Na(K)Cl_6_] and [InCl_6_] octahedra (hence STEs are more likely to migrate to the surface), rendering them more prone to non-radiative losses. In all these materials, the (large) Stokes shift was found to depend strongly on the lattice cage that surrounds the [SbCl_6_] octahedron. Our computational analysis on the origin of such shift found that in the DPs the large size of the K^+^ ion causes a more marked structural rearrangement in the excited state than the corresponding smaller Na^+^ ion, indicating that, by changing the type of B^+^ ions, the emission wavelength can be, in principle, tuned on-demand.

Motivated by their intense PL and large Stokes shifts, we tested our NCs as re-absorption-free scintillators for high-energy radiation detection. Both DP systems feature intense X-ray radioluminescence (RL), closely matching their respective PL, with negligible overlap with the corresponding absorption profile and nearly perfect radiation hardness for almost 500 Gy of cumulative delivered dose. The 0D NCs had instead a main RL at ∼4 eV (originating from the host lattice) overlapping with their absorption and were not studied further. The applicability of these DP systems in real re-absorption-free plastic scintillator detectors was finally simulated via Monte Carlo ray-tracing calculations, revealing essentially perfect waveguiding performance in large-area devices compatible with real-world applications. This result suggests a new, still unexplored materials design concept for Stokes shift engineering that exploits Jahn–Teller distortions upon photo-excitation of dopant-related emissive centers, with great potential for light management technologies based on wavelength-shifting waveguides.

The synthesis of Sb-doped DP NCs having a Sb content varying from 0 to 8.0% (atomic% with respect to In), as emerged from elemental analyses based on energy-dispersive spectroscopy in the scanning electron microscope (SEM-EDS) and on inductively coupled plasma optical emission spectroscopy (ICP-OES) (see Tables S1 and S2), is described in detail in the Supporting Information (SI). Our transmission electron microscopy (TEM) analysis indicated that the Sb-doped Cs_2_NaInCl_6_ NCs have a mean size around 20 nm (Figure 1a,b and Figure S1), while the Sb-doped Cs_2_KInCl_6_ NCs are smaller, with a mean diameter around 13 nm (Figure 1c,d and Figure S2). X-ray powder diffraction (XRD) characterization of the NC samples indicated that they all exhibit a DP cubic structure. Specifically, the XRD patterns of Cs_2_NaInCl_6_ NCs well match with the bulk DP Cs_2_NaInCl_6_ structure reported by Noculak et al.^46^ (Figure 1g), albeit with slightly larger lattice parameters (*a* = 10.533 Å in this work and *a* = 10.514 Å in the work of Noculak et al.; see Figure S3). Given the absence of known DP Cs_2_KInCl_6_ crystal structures, in the case of Cs_2_KInCl_6_ NCs we proceeded by employing the whole-powder-pattern decomposition technique, based on the Pawley algorithm,^57^ which indicated that these samples possess a cubic crystal structure (space group *Fm*3̅*m*) with the cell parameter *a* = 10.871 Å (Figure 1h and Figure S4). Compared to the bulk reflections of the DP Cs_2_NaInCl_6_ material, the diffraction peaks of Cs_2_KInCl_6_ NCs are slightly shifted to lower 2θ angles owing to the difference between the ionic radii of Na^+^ and K^+^ (102 pm for Na^+^ and 138 for K^+^). It is interesting to note here that bulk Cs_2_KInCl_6_ crystals tend to crystallize in a tetragonal structure,^46^ thus adopting a lower symmetry with respect to our cubic NCs. In both DP systems (Cs_2_NaInCl_6_ and Cs_2_KInCl_6_), the introduction of Sb did not result in a shift of the XRD peaks, most likely due to the fact that Sb^3+^ and In^3+^ cations have similar ionic radii (80 pm for In^3+^ and 76 pm for Sb^3+^).^58^

**Figure 1:**
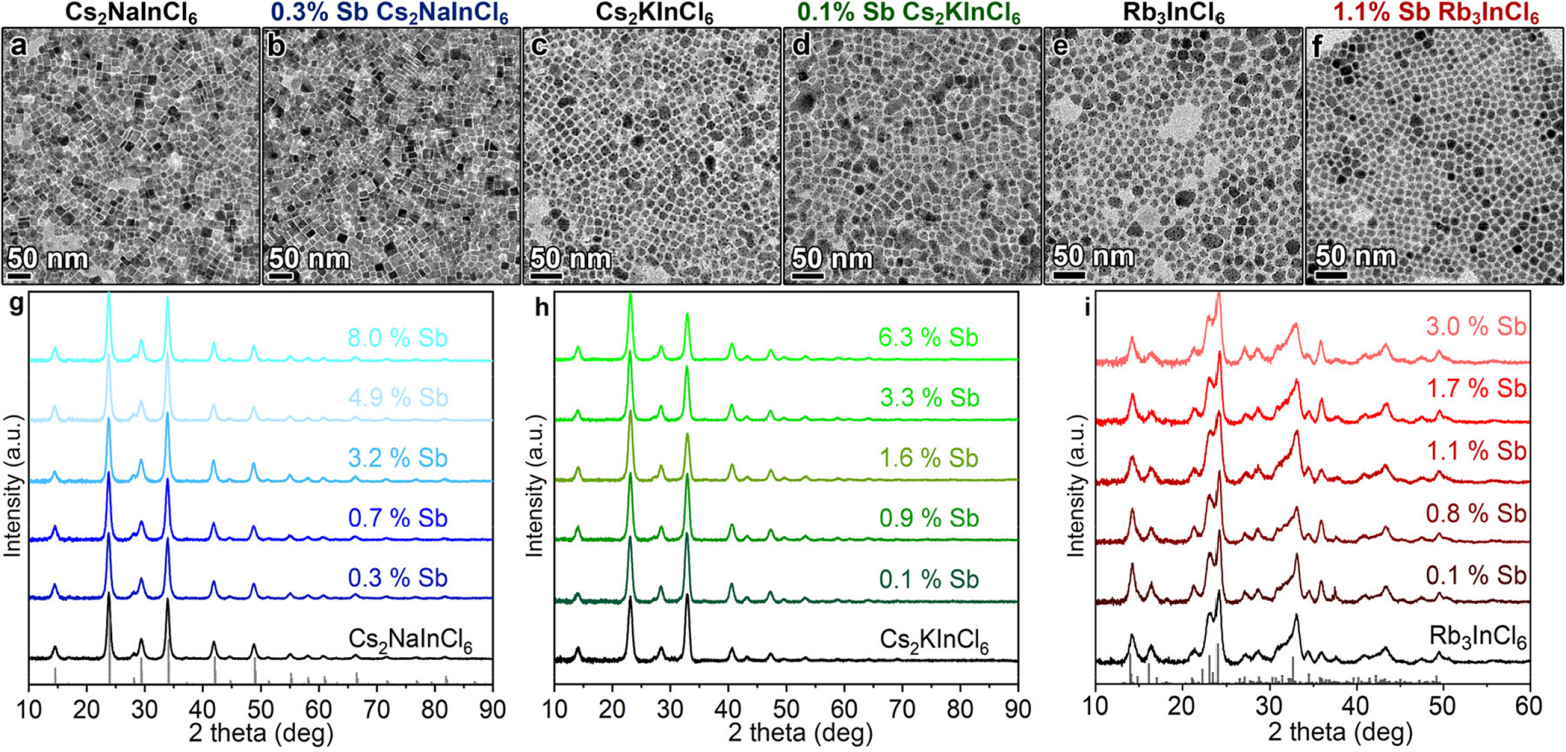
Bright-field TEM images of (a) undoped and (b) 0.3% Sb-doped Cs_2_NaInCl_6_ NCs; (c) undoped and (d) 0.1% Sb-doped Cs_2_KInCl_6_ NCs; and (e) undoped and (f) 1.1% Sb-doped Rb_3_InCl_6_ NCs. XRD patterns of (g) Cs_2_NaInCl_6_, (h) Cs_2_KInCl_6_, and (i) Rb_3_InCl_6_ NC samples containing different Sb doping amounts. The gray bars in (g) and (i) are the bulk reflections of the cubic DP Cs_2_NaInCl_6_ and Rb_3_TlCl_6_ (ICSD number 300228) crystal structures, respectively.

By adopting a similar synthesis strategy (again, see SI for details), we also prepared Sb-doped “0D” Rb_3_InCl_6_ NCs with a Sb content varying from 0 to 3.0% (atomic% with respect to In), as emerged from SEM-EDS and ICP-OES elemental analyses (Table S3). The size of these NCs was around 14 nm, as indicated by TEM (Figure 1e,f and Figure S5). The XRD pattern of such NCs could not be indexed with the monoclinic (space group *C*2/*c*) Rb_3_InCl_6_ structure recently reported by Majher et al.^52^ On the other hand, we found a good match with the monoclinic Rb_3_TlCl_6_ structure (ICSD no. 300228) belonging to the *P*121/*c*1 space group (Figure 1i). The XRD pattern of our NC samples was characterized by peaks that are slightly shifted toward higher 2θ angles with respect to those of Rb_3_TlCl_6_, consistent with the smaller ionic radius of In^3+^ compared with Tl^3+^ ions (88 pm for Tl^3+^ and 80 pm for In^3+^).^58^

To provide insights into the electronic structure of Sb-doped 3D and 0D systems, we performed density functional theory (DFT) calculations. Some of the works published so far have described the absorption and emission features of these systems as mostly atomic-like (i.e., ascribed to Sb ions only).^43−47,51−55^ Our assumption is that the [SbCl_6_] octahedron is electronically isolated in both materials: in the 0D by virtue of the crystal structure, and in the 3D because it is surrounded by six wide bandgap [B^+^Cl_6_] octahedra [B^+^ = Na^+^, K^+^]. We thus computed the electronic structure of the [SbCl_6_]^3–^ system with a local *O*_*h*_ symmetry which is found in both the DP and 0D lattices, with the Cs or Rb ions not participating to the band edges. With this small model system, we were able to include, in a simple way, also the spin–orbit coupling contribution, thus providing a clear understanding of the absorption features of the sensitizing centers, namely the [SbCl_6_] octahedra. In Figure 2, we show that the spin-free relativistic calculations at the DFT/PBE level of theory give a HOMO–LUMO gap of 3.76 eV, whereas the inclusion of spin–orbit coupling splits the t_1u_ triply degenerate LUMOs into doubly degenerate e_1/2u_ (3.46 eV) and quadruply degenerate u_3/2u_ (3.91 eV) molecular orbitals (MOs). Upon excitation from the e_1/2g_ doubly degenerate HOMO, 12 possible excitations are possible, as depicted on the right side of Figure 2. Here we labeled the transitions also according to the atomic symmetry, which helps to draw a comparison with an atomic-like description. The order of the transitions is the same as in the atomic case for Sb^3+^, although the presence of σ-antibonding interactions between the 5s of Sb and the 3p of Cl ions decreases dramatically the HOMO–LUMO gap by 5 eV and also reduces the differences among the states. Then, the transitions from the ground state to A_1u_ (^3^P_0_) and T_1u_ (^3^P_1_) have very low oscillator strengths because are mostly spin-forbidden (singlet to triplet), with selection rules that are only a little relaxed in the presence of spin–orbit coupling, suggesting that these systems will still retain long emission lifetimes.

**Figure 2:**
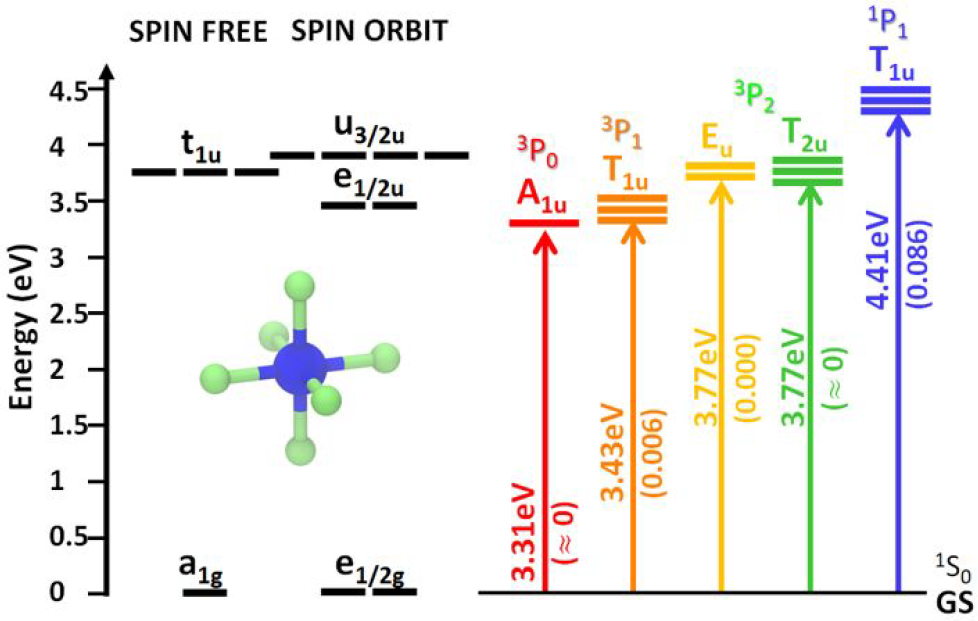
HOMO and LUMO levels of an ideal [SbCl_6_]^3–^ complex featuring an *O*_*h*_ symmetry and calculated at the DFT/PBE level of theory (left) using a spin-free relativistic approximation and (center) including the spin–orbit coupling effects. (right) Lowest spin–orbit coupled electronic transitions with the associated energies and oscillator strengths named according to the free ion (top) and the *O*_*h*_ symmetry (bottom).

On these grounds, we investigated the photophysics of the synthesized NCs via side-by-side optical spectroscopy experiments. In Figure 3a, we report the Tauc plot of the optical absorption spectrum of undoped Cs_2_NaInCl_6_ NCs, showing the clear signature of a direct forbidden band gap at 2.9 eV, in agreement with previous reports on bulk analogues.^13,43^ These NCs exhibit no appreciable PL, consistent with the very low oscillator strength of the intrinsic edge-to-edge transition.^59^ A similar optical behavior was seen in Cs_2_KInCl_6_ NCs, which had a slightly larger energy gap (3.0 eV, Figure 3b). The presence of Sb dopants in either DP host introduces two sharp features in the absorption profile at 3.90 and 3.72 eV (Figure 3c,d) and activates a broad, largely Stokes-shifted PL at 2.73 and 2.44 eV for the Sb-doped Cs_2_NaInCl_6_ and Cs_2_KInCl_6_ NCs, respectively. Notably, both the optical absorption profiles and the PL energies are essentially independent from the Sb content. This supports the hypothesis that the NCs’ photoexcitation and radiative relaxation processes are caused by transitions of individual [SbCl_6_] octahedra, in agreement with the STEs commonly invoked to explain the optical behavior of Sb-doped DPs bulk crystals. In emission, this effect would also account for the large Stokes shift connected with a Jahn–Teller lattice rearrangement (more details in the computational section in the SI).^43^

**Figure 3:**
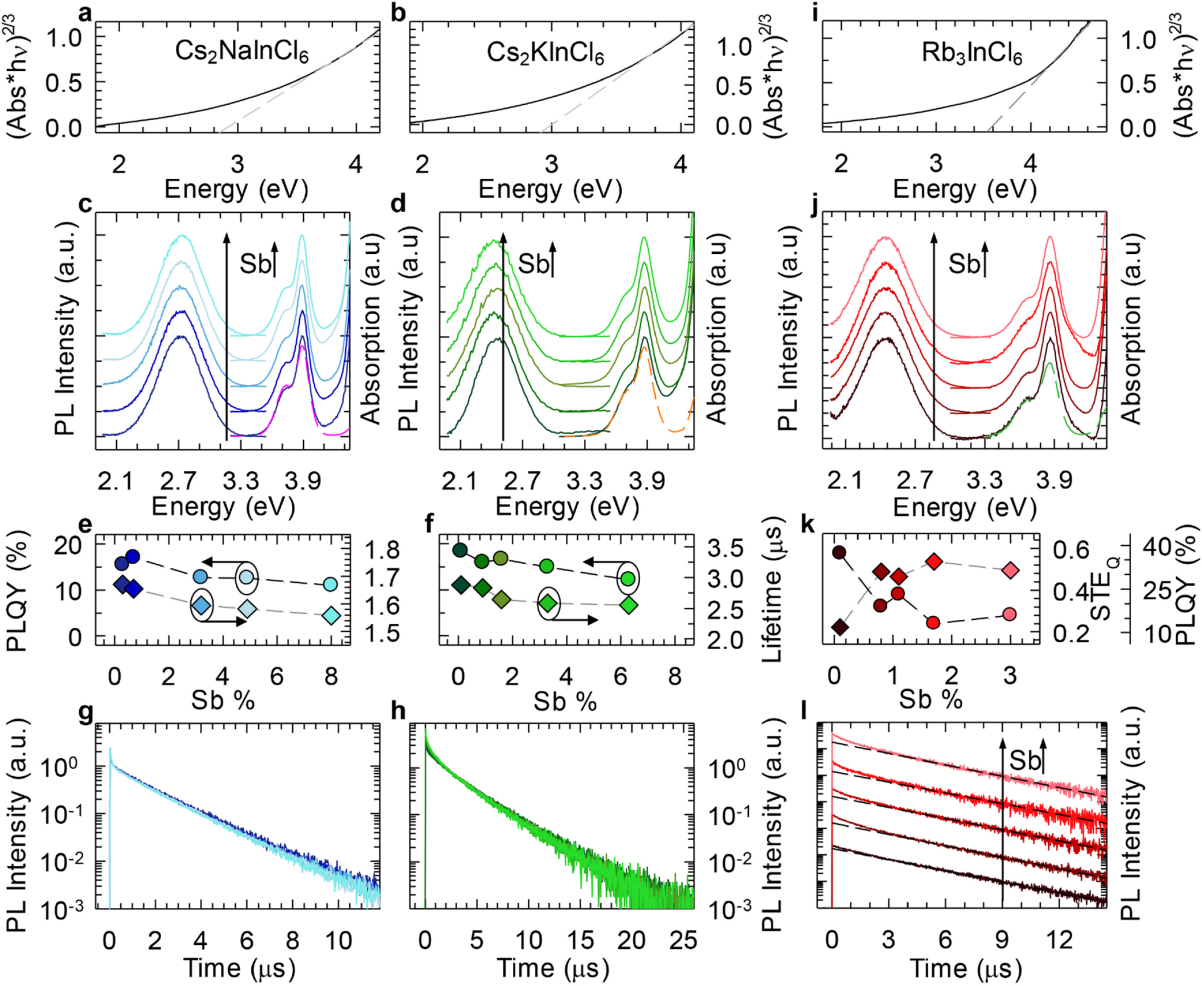
Tauc plot for undoped Cs_2_NaInCl_6_ (a) and Cs_2_KInCl_6_ (b) NCs. Gray dashed lines represent the fit with the theoretical absorption profile of a semiconductor with a direct forbidden energy gap; the intercept with the abscissa axis corresponds to the semiconductor’s forbidden gap energy. (c, d) Absorption and PL spectra for Sb-doped Cs_2_NaInCl_6_ (c) and Sb-doped Cs_2_KInCl_6_ (d) NCs with increasing Sb content, from bottom to top. PL excited with 3.87 eV (320 nm). Representative normalized PLEs obtained for PL maxima are reported as dashed lines. (e) Absolute PLQY (circles) and PL lifetimes (diamonds) extracted from (g) as a function of the incorporated Sb amount for Sb-doped Cs_2_NaInCl_6_ NCs. (f) Same as (e) for Sb-doped Cs_2_KInCl_6_ NCs. Lifetimes are extracted from (h). (g, h) Normalized PL decay after the fast initial drop (at 500 ns for (g) and 2 μs for (h)) using 3.49 eV (355 nm) pulsed excitation modulated at 2 kHz. (i) Tauc plot for the undoped Rb_3_InCl_6_ NCs. The gray dashed lines represent the fit with the theoretical absorption profile of a semiconductor with a direct forbidden energy gap; the intercept with the abscissa axis corresponds to the semiconductor’s forbidden gap energy. (j) Absorption and PL spectra for the set of Sb-doped Rb_3_InCl_6_ NCs with increasing Sb concentration from the bottom to the top. PL excited with 3.87 eV (320 nm). A representative normalized PLE obtained for PL maxima is reported as a green dashed line. (k) Absolute PLQY (circles) and the relative intensity of the STE_Q_ contribution (diamonds)—extracted from (l)—as a function of the Sb amount. (l) Set of normalized PL decays after the fast initial drop at 2 μs using 3.49 eV (355 nm) pulsed excitation modulated at 2 kHz. The Sb concentration increases from bottom to top. The black dashed lines are the fit with a single-exponential decay function excluding the first 3 μs.

The PL excitation (PLE) spectra collected at the PL maxima (dashed lines in Figure 3c,d) support this scenario by demonstrating that the PL originates from the radiative relaxation of SbCl_6_ excited states. The PLQY at different Sb contents is almost constant for both sets of DP NCs (Figure 3e,f), slightly decreasing from ∼15–18% for the lowest Sb doped sets of NCs to ∼11% for the highest doped set of NCs. This is consistent with the very weak dependence of the PL decay dynamics of either systems on the Sb content, as reported in Figure 3g,h. The PL decay dynamics evidence a long-lived single exponential luminescence decay with lifetimes of ∼1.6 μs for Sb-doped Cs_2_NaInCl_6_ and ∼2.7 μs for Sb-doped Cs_2_KInCl_6_ NCs. The initial very fast component (∼8 ns), common to the whole sets of NCs, is due to the PL from the surfactants under UV excitation. In both cases, the PL lifetime is compatible with a partially spin-forbidden transition undergoing a slight acceleration with increasing Sb content, in accordance with the respective PLQY trend (Figure 3e,f).

Notably, by comparing Sb-doped Cs_2_NaInCl_6_ and Cs_2_KInCl_6_ NCs, it appears that the latter exhibit slower recombination rates and are also characterized by an initial multi-exponential decay component which is consistent with a higher lattice disorder, induced, most likely, by a more pronounced structural distortion in the excited state. This, in turn, accounts for the larger Stokes shift between the absorption and PL spectra. A similar behavior has been reported by Nokulak et al. for bulk crystals of the same compositions.^46^

Having assessed the optical properties of the DPs NCs, we then studied the photophysics of undoped and Sb-doped “0D” Rb_3_InCl_6_ NCs. The undoped system shows an optical behavior similar to that of the DP NCs just discussed, with a direct forbidden band gap at 3.6 eV (Figure 3i) and no appreciable PL. The incorporation of Sb dopants introduces the characteristic double-absorption feature at 3.9 and 3.7 eV, whose optical excitation turns on the PL at 2.45 eV (Figure 3j). The PLE spectra confirm this picture, indicating that also in Sb-doped Rb_3_InCl_6_ NCs the transient distortion of the [SbCl_6_] octahedra is responsible for the large Stokes shift between the absorption and PL spectra and that the overall optical behavior is independent from the incorporated Sb amount. Overall, based also on DFT calculations (Figure 2), we conclude that the observed double-absorption peak characterizing both Sb-doped DP and Sb-doped 0D systems can be ascribed to the allowed transitions T_1u_ (^3^P_1_) at high energies (3.9 eV) and A_1u_ (^3^P_0_) at lower energies (3.7 eV). Notably, the computed oscillator strength for the lowest T_1,u_ (^3^P_1_) transition (*f* = 0.006) corresponds to a radiative lifetime of 0.2 μs, in fair agreement with the observed lifetimes. The 0D systems with the lowest Sb content exhibited a PLQY of ∼37%, nearly a factor of 2 higher than that of the DP NCs discussed earlier (Figure 3k). By increasing the Sb content, the PLQY decreased to ∼14%, which is comparable to the highest value found for the DP systems, in line with the gradual activation of a concentration quenching channel, bringing the excitation closer to quenching centers (Figure 3k). This picture is corroborated by time-resolved PL data (Figure 3l). Specifically, 0.1% Sb-doped NCs featured a nearly perfect single-exponential decay, with lifetime τ = 3.1 μs. This component remained unvaried with increasing the Sb content which, on the other hand, caused the gradual growth of a faster decay component with τ = 0.9 μs lifetime, ascribed to non-radiative quenching. Consistently, the relative weight of such a contribution (labeled STE_Q_) to the total PL decay nearly perfectly anti-correlates with the respective PLQY (Figure 3k), further supporting our hypothesis that such a kinetic effect is the signature of non-radiative quenching.

Overall, our optical analyses indicated that the optical features of both systems (DP and 0D) were very similar, with the main two differences being (i) the three sets of NCs studied (Cs_2_NaInCl_6_, Cs_2_KInCl_6_, and Rb_3_InCl_6_) all feature different Stokes shifts and (ii) the PLQYs of our NCs are always much lower than their bulk counterparts, however with the 0D NCs performing considerably better than the DPs. In order to explain such findings, we started to include in the computational model the whole lattice for both the 3D (Cs_2_NaInCl_6_ and Cs_2_KInCl_6_) and 0D (Rb_3_InCl_6_) systems, by applying a 2×2×2 replication of their unit cells. By substituting one In for one Sb, we obtained a doping concentration of 3% for the DPs and 2% for the 0D, in line with the experiments and previous works (see further details in the computational section in the SI). As also pointed out by Noculak et al.,^46^ and as illustrated in Figure 4a–c, the a_1g_ state of [SbCl_6_], composed of 5s orbitals of Sb, contributes to a MO localized at the valence band maximum (VBM), whereas the 5p’s of Sb contribute to the t_1u_ MOs that fall within the conduction band, whose minimum (CBM) is dominated by the In 5s orbitals. Despite this, and as anticipated earlier, the transitions involving In ions are dark, and the only optically active ones are those of Sb. This, however, raises a big challenge for DFT, because its variational nature makes it always collapsing to the lowest energetic state for a given spin multiplicity.

**Figure 4:**
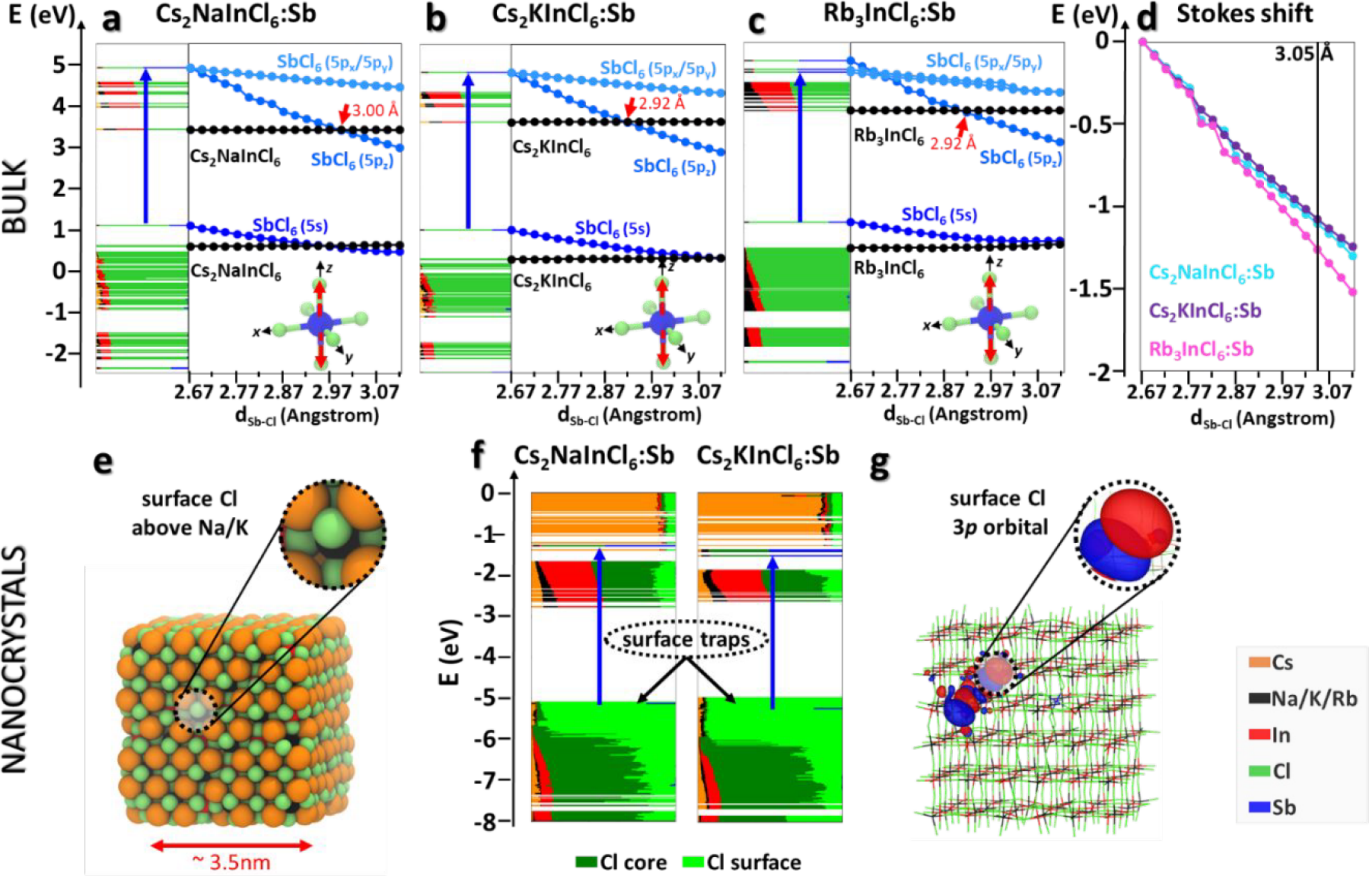
Electronic energy levels of (a) Cs_2_NaInCl_6_, (b) Cs_2_KInCl_6_, and (c) Rb_3_InCl_6_ (left) 2×2×2 supercells doped with one Sb^3+^ ion computed at the Γ point at the DFT/PBE level of theory. Each orbital is represented in real space and decomposed according to the participating atom types. The systematic elongation of the Sb–Cl bond is shown near each energy level plot. Here, the CBM and VBM of the [InCl_6_] matrix are depicted in black, whereas those of SbCl_6_ are in blue. (d) Stokes shift energies of the lowest electronic transition involving the SbCl_6_ octahedron upon systematic stretching of the axial Sb–Cl bonds in the Sb-doped (cyan) Cs_2_NaInCl_6_, (purple) Cs_2_KInCl_6_, and (magenta) Rb_3_InCl_6_ supercells. The equilibrium length of the axial bond (estimated around 3.05 Å) is indicated by the black vertical line. (e) Ball representation of a cubic Sb-doped Cs_2_AInCl_6_ [A = Na, K] NC model of about 3.5 nm in size, optimized at the DFT/PBE level of theory. Inset: Coordination of a surface Cl above the A cation. (f) Electronic energy levels of Cs_2_NaInCl_6_ and Cs_2_KInCl_6_ models, indicating a relevant contribution of Cl ions (light green) to trap states above the valence band. (g) The orbital density plot reveals a strong localization of these states at the NC surface, and more specifically on the Cl 3p orbitals (inset).

To overcome this problem, we decided to qualitatively follow the electronic structure of both the 3D and 0D systems while systematically elongating the axial Sb–Cl bonds, i.e., the bond that is partially broken upon photoexcitation, as it involves the occupation of just one of the three-fold-degenerate 5p(Sb)–3p(Cl) antibonding molecular orbitals. As illustrated in Figure 4a–c, this orbital goes down faster than the occupied orbital involving the Sb(5s), and it ultimately accounts for the Jahn–Teller distortion, i.e., the rupture of the t_1u_ degeneracy, and, thus, is also responsible for the measured Stokes shift. Notably, considering that the equilibrium axial bond length is expected at about 3.05 Å (estimated on the Sb-doped “0D” Rb_3_InCl_6_ system; see SI), we could extrapolate Stokes shift energies in the range of 1.1–1.3 eV (Figure 4d), in agreement with the experiments, but with the difference that the computed Stokes shift of Cs_2_NaInCl_6_ is about the same as or even larger than that of Cs_2_KInCl_6_. To explain this discrepancy with the experiments, we speculate that the larger Stokes shift observed for the K case could also stem from secondary distortion effects, like octahedron tilting, because the lattice cannot accommodate a full stretch of the Sb–Cl bond due to the larger size of K ions compared to Na.

With regard to the PLQY of our Sb-doped NC samples, they exhibit a moderate PL efficiency if compared to their bulk counterparts, as already observed in the case of bulk and nano Bi-doped Cs_2_(Ag,Na)InCl_6_ DP systems.^39,46,52,54^ This difference has been attributed to the incomplete surface passivation of synthesized colloidal NCs, hence to the presence of under-coordinated ions at their surface, which can lead to the emergence of mid-gap electronic states.^39^ To achieve a more realistic description of our systems that explicitly accounts for these surface effects, we carried out a DFT investigation on cubic NC models in line with the experiments (Figure 4e and computational details). Note that we failed to prepare an atomistic model for the 0D system due to an overall low symmetry that made it unclear how to terminate the surface. We however expect that the source of traps for the 0D is of the same nature as in the 3D materials. Even when the surface is fully passivated, the lower coordination of the surface chlorine ions (light green in Figure 4f), compared to those located in the core (dark green), determines the destabilization of their 3p orbitals, which are accordingly pushed toward the top of the valence band and are strongly localized (Figure 4g). This effect is particularly pronounced for those surface Cl anions that sit on top of Na/K cations (inset of Figure 4e), since the latter act as efficient electron barriers, thus preventing any delocalization—hence stabilization—of the corresponding Cl states through the NC. Consequently, these states emerge above the 5s-based state of the SbCl_6_ emission center, therefore potentially behaving as hole traps in Sb-doped Cs_2_NaInCl_6_ and Cs_2_KInCl_6_ NCs.

With these arguments at hand, we can hypothesize that the photo-generated holes are transported from the SbCl_6_ centers to the surface within the emission lifetime, thus providing an efficient channel of non-radiative recombination. While a precise estimate of the density of surface defects characterizing these systems would be extremely challenging, our optical analyses suggest that both 0D and 3D NCs could be characterized by a similar density of trap states, since at high doping levels the PLQY values for all systems tend to similar values. The visible difference in PLQY between the 0D and the 3D NC systems at low doping levels can then be ascribed to the fact that, in the 0D NCs, the hole carrier has to hop from one octahedron to another in order to reach a surface trap state, whereas in the 3D NCs the connection between octahedra enhances the electron–phonon coupling effect via lattice vibrations, thus accelerating the non-radiative decay. In 0D NC systems, at higher doping levels the probability of a dopant to be closer to the NC’s surface is statistically higher, meaning a higher chance for the STEs to reach a trap state, and thus to contribute to an overall lower PLQY. Also, we can speculate that an increase in NC size would not only lower the surface/volume ratio, thus the overall density of defect states, but also the probability of STEs reaching such traps, attaining eventually the PLQY values characterizing bulk systems.

To test the potential suitability of both Sb-doped DP and 0D perovskite NCs as re-absorption-free scintillator materials, we tested their radioluminescence (RL) emission and stability under prolonged continuous irradiation with soft X-rays and calculated the waveguiding properties of plastic scintillators embedding such NCs using Monte Carlo ray-tracing simulations. The RL spectra for 0.7% Sb-doped Cs_2_NaInCl_6_ and 0.9% Sb-doped Cs_2_KInCl_6_ NCs were dominated by peaks at ∼2.7 and ∼2.4 eV, respectively, which very well resembled the respective PL emissions (Figure 5a,c), thus indicating that the interaction with ionizing radiation creates the same excited states as UV optical excitation. Interestingly, the RL evidenced two additional minor spectral contributions at ∼2.1 and ∼4.2 eV, common to both samples, which we attribute to the DP host matrix and possibly originating from the [InCl_6_] octahedra present in both DP structures. The observation of such signals confirms the particular sensitivity of the RL technique to detect even minority emissions that are generally overlooked in PL experiments. The RL stability of these materials is presented in Figure 5b,d and Figure S6. For these experiments, the NC samples were exposed to continuous X-ray irradiation (dose rate of 0.5 Gy/s) up to almost 500 Gy of cumulated delivered dose. Remarkably, the RL intensities retained their initial value while the accumulated dose was increased, suggesting the absence of either competitive shallow traps for the Sb emissive excited states or damage to the NCs under prolonged exposure to X-rays. In stark contrast to the 3D DP systems, the RL spectrum of 0D 0.8% Sb-doped Rb_3_InCl_6_ NCs in Figure 5e was dominated by the host emissions at ∼2 and ∼4.2 eV (dashed gray line), while the Sb-related RL at 2.45 eV gave a minor contribution. Also in this case, the prolonged exposure to X-rays did not affect the RL intensity (Figure 5f).

**Figure 5:**
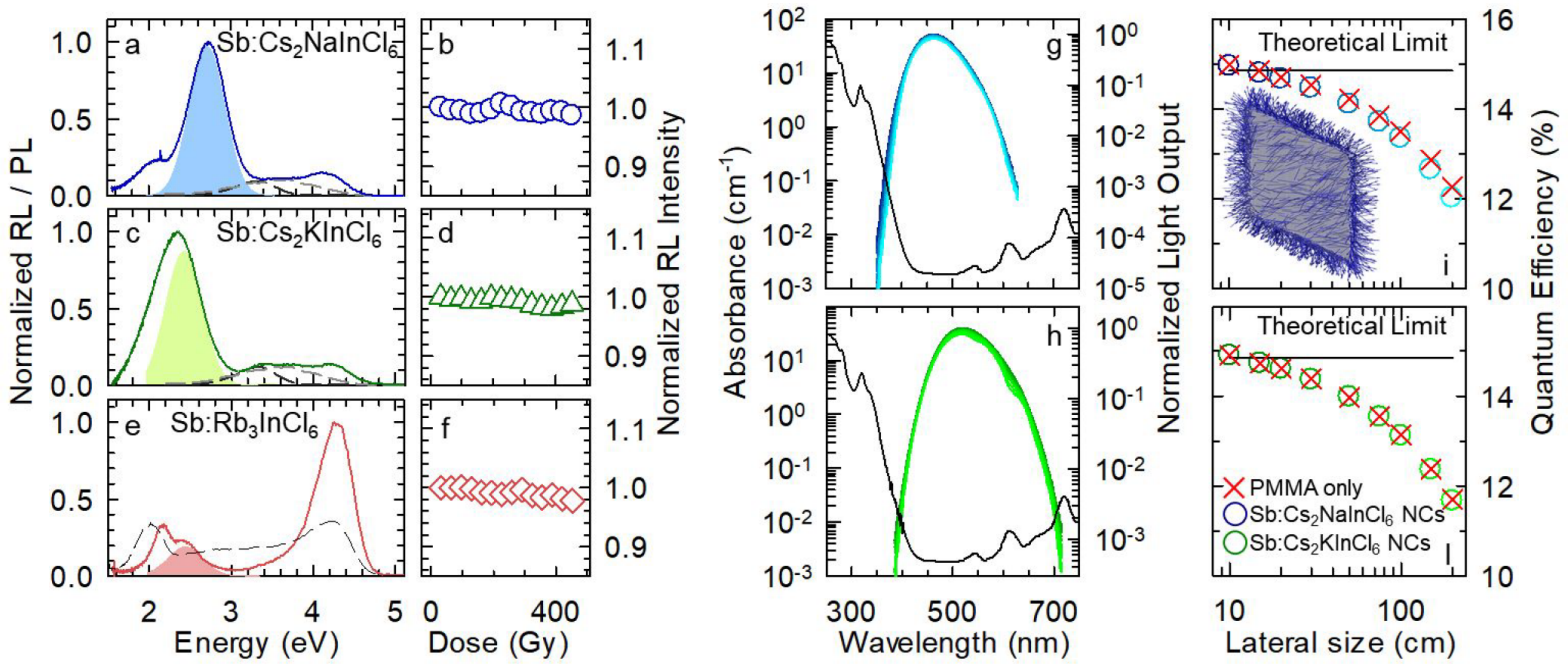
RL spectra (solid lines) at room temperature excited using soft X-rays for representative samples of (a) 0.7% Sb-doped Cs_2_NaInCl_6_, (c) 0.9% Sb-doped Cs_2_KInCl_6_, and (e) 0.8% Sb-doped Rb_3_InCl_6_ NCs. The corresponding PL spectra from Figure 3 are shown as shaded curves. In (a) and (c), the RL spectra of oleic acid and oleylamine are shown as black and gray curves, respectively. The dashed gray line in (e) is the RL spectrum of undoped Rb_3_InCl_6_ NCs. (b, d, f) Corresponding integrated RL intensities at increasing cumulative dose up to 500 Gy, with a dose rate of 0.5 Gy/s. Data are normalized to the initial value, and each data point represents the integral of the RL emission spectrum in the 1.8–3.0 eV range. Monte Carlo ray-tracing simulation of the RL spectra as a function of increasing device size (10 × 10 × 1 cm^3^ to 2 × 2 m^2^ × 1 cm, 10 wt%, from dark to light colored lines) calculated considering the experimental PLQY of 20% of (g) 0.7% Sb-doped Cs_2_NaInCl_6_ and (h) 0.9% Sb-doped Cs_2_KInCl_6_ NCs. (i, l) Respective spectrally integrated RL intensities (circles) vs device size showing near-perfect coincidence with the trend, considering solely the re-absorption effect by the PMMA matrix (red crosses). Inset in (i): Visualization of Monte Carlo ray-tracing simulation for a plastic scintillator embedding Sb-doped Cs_2_NaInCl_6_.

Overall, our RL analyses indicate that only Sb-doped DPs NCs are promising candidates for scintillation applications, since most of their RL emission does not overlap with their absorption. We therefore simulated the waveguiding performance and expected quantum efficiency of square nanocomposite plastic scintillators (1 cm thick, lateral size from 10 cm × 10 cm to 2 m × 2 m) made of poly(methyl methacrylate) (PMMA) embedding 10 wt% of Cs_2_NaInCl_6_ or Cs_2_KInCl_6_ NCs doped with Sb (0.7% and 0.9%, respectively). For the calculations, we used an emission quantum yield of 20%, consistent with the measured PLQY, and PMMA was chosen for its substantially higher transparency in the visible spectrum with respect to any other available polymer processable by the cell-casting method, which is necessary for producing optical-grade plastic waveguides.^60^ The absorption spectra of the nanocomposites (computed along the waveguide thickness) are reported in Figure 5g,h, showing, in both cases, the absorption edge of the NCs below ∼420 nm and the absorption overtones of the vibrational modes of the polymer matrix above 540 nm. The RL spectra emitted by the edges of the two scintillators are also reported for increasing device size (Figure 5g,h, dark to light colored lines), showing, for both NC systems, very small spectral overlap with the respective low-energy tail of the absorption spectrum. This results in negligible losses, due to re-absorption by the NCs, as quantified in Figure 5i,l, where we report the evolution of the device quantum efficiency (QE, evaluated as the number of photons emitted from the waveguide edges divided by the number of excitons generated inside the device upon exposure to high-energy radiation) with the waveguide size. In order to decouple NC and matrix effects, the same figure also shows the trend of the QE, considering exclusively the re-absorption of the propagating RL by PMMA, and the theoretical limit for waveguides embedding the same NCs content, but neglecting matrix effects (Figure 5i,l). Interestingly, for any scintillator size, the QE of the actual device (where the effect of both the NCs and the matrix is taken into account) closely matches the case considering exclusively PMMA, thus confirming the nearly perfect suppression of re-absorption in these DP systems. We further notice that, while K-based NCs suffer even lower re-absorption losses than the Na-based counterparts, the overall waveguiding properties of the two devices are nearly identical due to the stronger resonance between the RL of the Sb:Cs_2_KInCl_6_ NCs and the low-energy vibrational modes of the matrix. In either case, such a loss may be further suppressed by using fluorinated polymers.^60^

In conclusion, we have reported the syntheses of colloidal nanocrystals of Rb_3_InCl_6_ (0D) metal halide and of Cs_2_NaInCl_6_ and Cs_2_KInCl_6_ (3D) double perovskites and their doping with Sb^3+^ cations. All the Sb-doped nanocrystal systems show consistently lower PL than their bulk counterparts, a behavior that we ascribed to deep trap states originated by surface under-coordinated halide ions. Sb-doped “0D” Rb_3_InCl_6_ nanocrystals exhibit a higher PLQY compared to the Sb-doped “3D” Cs_2_NaInCl_6_ and Cs_2_KInCl_6_ ones. We attribute this finding to the different connectivity of metal halide octahedra characterizing 0D and 3D structures: isolated octahedra in the 0D structure reduce the exciton diffusion, thus minimizing non-radiative decay. The applicability of the systems as scintillator materials is assessed via RL measurements and corroborated by Monte Carlo ray-tracing simulations, highlighting their potential for re-absorption-free plastic scintillators of very large size. The excellent waveguiding capability found for these DP nanocrystals is particularly important for any light management technology based on wavelength-shifting waveguides and suggests a new, still unexplored route for Stokes shift engineering exploiting Jahn–Teller distortion upon photo-excitation. This study demonstrates once again that doping of various nanoscale metal halide systems can uncover interesting physics and deliver materials that can be useful in technological applications. The surface remains of paramount importance in all these systems, due to the much reduced tolerance compared to lead halide perovskites, and future synthetic strategies should aim to shore up this issue, for example, with proper ligand functionalization and/or growth of a large-bandgap inorganic shell.

## Supporting Information Available

Experimental details, supplementary TEM pictures, SEM and ICP analyses, and whole-powder-pattern decomposition (PDF)

## References

[ref1] Yuan M.; Quan L. N.; Comin R.; Walters G.; Sabatini R.; Voznyy O.; Hoogland S.; Zhao Y.; Beauregard E. M.; Kanjanaboos P.; Lu Z.; Kim D. H.; Sargent E. H. Perovskite Energy Funnels for Efficient Light-Emitting Diodes. Nat. Nanotechnol. 2016, 11, 872–877. 10.1038/nnano.2016.110. 27347835

[ref2] Akkerman Q. A.; Rainò G.; Kovalenko M. V.; Manna L. Genesis, Challenges and Opportunities for Colloidal Lead Halide Perovskite Nanocrystals. Nat. Mater. 2018, 17, 394–405. 10.1038/s41563-018-0018-4. 29459748

[ref3] He X.; Qiu Y.; Yang S. Fully-Inorganic Trihalide Perovskite Nanocrystals: A New Research Frontier of Optoelectronic Materials. Adv. Mater. 2017, 29, 1700775 10.1002/adma.201700775. 28639413

[ref4] Luo J.; Wang X.; Li S.; Liu J.; Guo Y.; Niu G.; Yao L.; Fu Y.; Gao L.; Dong Q.; Zhao C.; Leng M.; Ma F.; Liang W.; Wang L.; Jin S.; Han J.; Zhang L.; Etheridge J.; Wang J.; Yan Y.; Sargent E. H.; Tang J. Efficient and Stable Emission of Warm-White Light from Lead-Free Halide Double Perovskites. Nature 2018, 563, 541–545. 10.1038/s41586-018-0691-0. 30405238

[ref5] Shamsi J.; Urban A. S.; Imran M.; De Trizio L.; Manna L. Metal Halide Perovskite Nanocrystals: Synthesis, Post-Synthesis Modifications, and Their Optical Properties. Chem. Rev. 2019, 119, 3296–3348. 10.1021/acs.chemrev.8b00644. 30758194 PMC6418875

[ref6] Song Z.; Shrestha N.; Watthage S. C.; Liyanage G. K.; Almutawah Z. S.; Ahangharnejhad R. H.; Phillips A. B.; Ellingson R. J.; Heben M. J. Impact of Moisture on Photoexcited Charge Carrier Dynamics in Methylammonium Lead Halide Perovskites. J. Phys. Chem. Lett. 2018, 9, 6312–6320. 10.1021/acs.jpclett.8b02595. 30336064

[ref7] Leng M.; Chen Z.; Yang Y.; Li Z.; Zeng K.; Li K.; Niu G.; He Y.; Zhou Q.; Tang J. Lead-Free, Blue Emitting Bismuth Halide Perovskite Quantum Dots. Angew. Chem., Int. Ed. 2016, 55, 15012–15016. 10.1002/anie.201608160. 27791304

[ref8] Filip M. R.; Hillman S.; Haghighirad A. A.; Snaith H. J.; Giustino F. Band Gaps of the Lead-Free Halide Double Perovskites Cs_2_BiAgCl_6_ and Cs_2_BiAgBr_6_ from Theory and Experiment. J. Phys. Chem. Lett. 2016, 7, 2579–2585. 10.1021/acs.jpclett.6b01041. 27322413

[ref9] Vasala S.; Karppinen M. A_2_B′B″O_6_ Perovskites: A Review. Prog. Solid State Chem. 2015, 43, 1–36. 10.1016/j.progsolidstchem.2014.08.001.

[ref10] Volonakis G.; Haghighirad A. A.; Milot R. L.; Sio W. H.; Filip M. R.; Wenger B.; Johnston M. B.; Herz L. M.; Snaith H. J.; Giustino F. Cs_2_InAgCl_6_: A New Lead-Free Halide Double Perovskite with Direct Band Gap. J. Phys. Chem. Lett. 2017, 8, 772–778. 10.1021/acs.jpclett.6b02682. 28133967

[ref11] Volonakis G.; Filip M. R.; Haghighirad A. A.; Sakai N.; Wenger B.; Snaith H. J.; Giustino F. Lead-Free Halide Double Perovskites Via Heterovalent Substitution of Noble Metals. J. Phys. Chem. Lett. 2016, 7, 1254–1259. 10.1021/acs.jpclett.6b00376. 26982118

[ref12] Zhou J.; Xia Z.; Molokeev M. S.; Zhang X.; Peng D.; Liu Q. Composition Design, Optical Gap and Stability Investigations of Lead-Free Halide Double Perovskite Cs_2_AgInCl_6_. J. Mater. Chem. A 2017, 5, 15031–15037. 10.1039/C7TA04690A.

[ref13] Meng W.; Wang X.; Xiao Z.; Wang J.; Mitzi D. B.; Yan Y. Parity-Forbidden Transitions and Their Impact on the Optical Absorption Properties of Lead-Free Metal Halide Perovskites and Double Perovskites. J. Phys. Chem. Lett. 2017, 8, 2999–3007. 10.1021/acs.jpclett.7b01042. 28604009

[ref14] Xuan T.; Xie R.-J. Recent Processes on Light-Emitting Lead-Free Metal Halide Perovskites. Chem. Eng. J. 2020, 393, 124757 10.1016/j.cej.2020.124757.

[ref15] Pham H. Q.; Holmes R. J.; Aydil E. S.; Gagliardi L. Lead-Free Double Perovskites Cs_2_InCuCl_6_ and (CH_3_NH_3_)_2_InCuCl_6_: Electronic, Optical, and Electrical Properties. Nanoscale 2019, 11, 11173–11182. 10.1039/C9NR01645G. 31149693

[ref16] Lv K.; Qi S.; Liu G.; Lou Y.; Chen J.; Zhao Y. Lead-Free Silver-Antimony Halide Double Perovskite Quantum Dots with Superior Blue Photoluminescence. Chem. Commun. 2019, 55, 14741–14744. 10.1039/C9CC07397C. 31754680

[ref17] Dahl J. C.; Osowiecki W. T.; Cai Y.; Swabeck J. K.; Bekenstein Y.; Asta M.; Chan E. M.; Alivisatos A. P. Probing the Stability and Band Gaps of Cs_2_AgInCl_6_ and Cs_2_AgSbCl_6_ Lead-Free Double Perovskite Nanocrystals. Chem. Mater. 2019, 31, 3134–3143. 10.1021/acs.chemmater.8b04202.

[ref18] Zhou J.; Rong X.; Zhang P.; Molokeev M. S.; Wei P.; Liu Q.; Zhang X.; Xia Z. Manipulation of Bi^3+^/In^3+^ Transmutation and Mn^2+^-Doping Effect on the Structure and Optical Properties of Double Perovskite Cs_2_NaBi_1-x_In_x_Cl_6_. Adv. Opt. Mater. 2019, 7, 1801435 10.1002/adom.201801435.

[ref19] Zhang Y.; Shah T.; Deepak F. L.; Korgel B. A. Surface Science and Colloidal Stability of Double-Perovskite Cs_2_AgBiBr_6_ Nanocrystals and Their Superlattices. Chem. Mater. 2019, 31, 7962–7969. 10.1021/acs.chemmater.9b02149.

[ref20] Zhang L.; Fang Y.; Sui L.; Yan J.; Wang K.; Yuan K.; Mao W. L.; Zou B. Tuning Emission and Electron-Phonon Coupling in Lead-Free Halide Double Perovskite Cs_2_AgBiCl_6_ under Pressure. ACS Energy Lett. 2019, 4, 2975–2982. 10.1021/acsenergylett.9b02155.

[ref21] Locardi F.; Cirignano M.; Baranov D.; Dang Z.; Prato M.; Drago F.; Ferretti M.; Pinchetti V.; Fanciulli M.; Brovelli S.; De Trizio L.; Manna L. Colloidal Synthesis of Double Perovskite Cs_2_AgInCl_6_ and Mn-Doped Cs_2_AgInCl_6_ Nanocrystals. J. Am. Chem. Soc. 2018, 140, 12989–12995. 10.1021/jacs.8b07983. 30198712 PMC6284204

[ref22] Creutz S. E.; Crites E. N.; De Siena M. C.; Gamelin D. R. Colloidal Nanocrystals of Lead-Free Double-Perovskite (Elpasolite) Semiconductors: Synthesis and Anion Exchange to Access New Materials. Nano Lett. 2018, 18, 1118–1123. 10.1021/acs.nanolett.7b04659. 29376378

[ref23] Giustino F.; Snaith H. J. Toward Lead-Free Perovskite Solar Cells. ACS Energy Lett. 2016, 1, 1233–1240. 10.1021/acsenergylett.6b00499.

[ref24] Igbari F.; Wang Z.-K.; Liao L.-S. Progress of Lead-Free Halide Double Perovskites. Adv. Energy Mater. 2019, 9, 1803150 10.1002/aenm.201803150.

[ref25] Ghosh S.; Pradhan B. Lead-Free Metal Halide Perovskite Nanocrystals: Challenges, Applications, and Future Aspects. ChemNanoMat 2019, 5, 300–312. 10.1002/cnma.201800645.

[ref26] Lamba R. S.; Basera P.; Bhattacharya S.; Sapra S. Band Gap Engineering in Cs_2_(Na_x_Ag_1-x_)BiCl_6_ Double Perovskite Nanocrystals. J. Phys. Chem. Lett. 2019, 10, 5173–5181. 10.1021/acs.jpclett.9b02168. 31415179

[ref27] Tran T. T.; Panella J. R.; Chamorro J. R.; Morey J. R.; McQueen T. M. Designing Indirect-Direct Bandgap Transitions in Double Perovskites. Mater. Horiz. 2017, 4, 688–693. 10.1039/C7MH00239D.

[ref28] Luo J.; Li S.; Wu H.; Zhou Y.; Li Y.; Liu J.; Li J.; Li K.; Yi F.; Niu G.; Tang J. Cs_2_AgInCl_6_ Double Perovskite Single Crystals: Parity Forbidden Transitions and Their Application for Sensitive and Fast Uv Photodetectors. ACS Photonics 2018, 5, 398–405. 10.1021/acsphotonics.7b00837.

[ref29] Zhao F.; Song Z.; Zhao J.; Liu Q. Double Perovskite Cs_2_AgInCl_6_:Cr^3+^: Broadband and Near-Infrared Luminescent Materials. Inorg. Chem. Front. 2019, 6, 3621–3628. 10.1039/C9QI00905A.

[ref30] Dave K.; Fang M. H.; Bao Z.; Fu H. T.; Liu R. S. Recent Developments in Lead-Free Double Perovskites: Structure, Doping, and Applications. Chem. - Asian J. 2020, 15, 242–252. 10.1002/asia.201901510. 31794155

[ref31] Zhao X.-G.; Yang D.; Ren J.-C.; Sun Y.; Xiao Z.; Zhang L. Rational Design of Halide Double Perovskites for Optoelectronic Applications. Joule 2018, 2, 1662–1673. 10.1016/j.joule.2018.06.017.

[ref32] Chen N.; Cai T.; Li W.; Hills-Kimball K.; Yang H.; Que M.; Nagaoka Y.; Liu Z.; Yang D.; Dong A.; Xu C. Y.; Zia R.; Chen O. Yb- and Mn-Doped Lead-Free Double Perovskite Cs_2_AgBiX_6_ (X = Cl^–^, Br^–^) Nanocrystals. ACS Appl. Mater. Interfaces 2019, 11, 16855–16863. 10.1021/acsami.9b02367. 30985112

[ref33] Liu Y.; Jing Y.; Zhao J.; Liu Q.; Xia Z. Design Optimization of Lead-Free Perovskite Cs_2_AgInCl_6_:Bi Nanocrystals with 11.4% Photoluminescence Quantum Yield. Chem. Mater. 2019, 31, 3333–3339. 10.1021/acs.chemmater.9b00410.

[ref34] Lee W.; Hong S.; Kim S. Colloidal Synthesis of Lead-Free Silver-Indium Double-Perovskite Cs_2_AgInCl_6_ Nanocrystals and Their Doping with Lanthanide Ions. J. Phys. Chem. C 2019, 123, 2665–2672. 10.1021/acs.jpcc.8b12146.

[ref35] Mahor Y.; Mir W. J.; Nag A. Synthesis and near-Infrared Emission of Yb-Doped Cs_2_AgInCl_6_ Double Perovskite Microcrystals and Nanocrystals. J. Phys. Chem. C 2019, 123, 15787–15793. 10.1021/acs.jpcc.9b02456.

[ref36] Du K.-z.; Meng W.; Wang X.; Yan Y.; Mitzi D. B. Bandgap Engineering of Lead-Free Double Perovskite Cs_2_AgBiBr_6_ through Trivalent Metal Alloying. Angew. Chem., Int. Ed. 2017, 56, 8158–8162. 10.1002/anie.201703970. 28523742

[ref37] Manna D.; Das T. K.; Yella A. Tunable and Stable White Light Emission in Bi^3+^-Alloyed Cs_2_AgInCl_6_ Double Perovskite Nanocrystals. Chem. Mater. 2019, 31, 10063–10070. 10.1021/acs.chemmater.9b02973.

[ref38] Gray M. B.; Majher J. D.; Strom T. A.; Woodward P. M. Broadband White Emission in Cs_2_AgIn_1-x_Bi_x_Cl_6_ Phosphors. Inorg. Chem. 2019, 58, 13403–13410. 10.1021/acs.inorgchem.9b02299. 31549818

[ref39] Zhang B.; Wang M.; Ghini M.; Melcherts A. E. M.; Zito J.; Goldoni L.; Infante I.; Guizzardi M.; Scotognella F.; Kriegel I.; De Trizio L.; Manna L. Colloidal Bi-Doped Cs_2_Ag_1-x_Na_x_InCl_6_ Nanocrystals: Undercoordinated Surface Cl Ions Limit Their Light Emission Efficiency. ACS Mater. Lett. 2020, 2, 1442–1449. 10.1021/acsmaterialslett.0c00359. 33644762 PMC7901666

[ref40] Lee W.; Choi D.; Kim S. Colloidal Synthesis of Shape-Controlled Cs_2_NaBiX_6_ (X = Cl, Br) Double Perovskite Nanocrystals: Discrete Optical Transition by Non-Bonding Characters and Energy Transfer to Mn Dopants. Chem. Mater. 2020, 32, 6864–6874. 10.1021/acs.chemmater.0c01315.

[ref41] Locardi F.; Sartori E.; Buha J.; Zito J.; Prato M.; Pinchetti V.; Zaffalon M. L.; Ferretti M.; Brovelli S.; Infante I.; De Trizio L.; Manna L. Emissive Bi-Doped Double Perovskite Cs_2_Ag_1-x_Na_x_InCl_6_ Nanocrystals. ACS Energy Lett. 2019, 4, 1976–1982. 10.1021/acsenergylett.9b01274.

[ref42] Yang B.; Mao X.; Hong F.; Meng W.; Tang Y.; Xia X.; Yang S.; Deng W.; Han K. Lead-Free Direct Band Gap Double-Perovskite Nanocrystals with Bright Dual-Color Emission. J. Am. Chem. Soc. 2018, 140, 17001–17006. 10.1021/jacs.8b07424. 30452250

[ref43] Zeng R.; Zhang L.; Xue Y.; Ke B.; Zhao Z.; Huang D.; Wei Q.; Zhou W.; Zou B. Highly Efficient Blue Emission from Self-Trapped Excitons in Stable Sb^3+^-Doped Cs_2_NaInCl_6_ Double Perovskites. J. Phys. Chem. Lett. 2020, 11, 2053–2061. 10.1021/acs.jpclett.0c00330. 32105076

[ref44] Wu S.; Li W.; Hu J.; Gao P. Antimony Doped Lead-Free Double Perovskites (Cs_2_NaBi_1-x_Sb_x_Cl_6_) with Enhanced Light Absorption and Tunable Emission. J. Mater. Chem. C 2020, 8, 13603–13611. 10.1039/D0TC03003A.

[ref45] Gray M. B.; Hariyani S.; Strom T. A.; Majher J. D.; Brgoch J.; Woodward P. M. High-Efficiency Blue Photoluminescence in the Cs_2_NaInCl_6_:Sb^3+^ Double Perovskite Phosphor. J. Mater. Chem. C 2020, 8, 6797–6803. 10.1039/D0TC01037E.

[ref46] Noculak A.; Morad V.; McCall K. M.; Yakunin S.; Shynkarenko Y.; Wörle M.; Kovalenko M. V. Bright Blue and Green Luminescence of Sb(III) in Double Perovskite Cs_2_MInCl_6_ (M = Na, K) Matrices. Chem. Mater. 2020, 32, 5118–5124. 10.1021/acs.chemmater.0c01004. 32595266 PMC7315817

[ref47] McCall K. M.; Morad V.; Benin B. M.; Kovalenko M. V. Efficient Lone-Pair-Driven Luminescence: Structure-Property Relationships in Emissive 5s^2^ Metal Halides. ACS Mater. Lett. 2020, 2, 1218–1232. 10.1021/acsmaterialslett.0c00211. 32954359 PMC7491574

[ref48] Liu X.; Xu X.; Li B.; Yang L.; Li Q.; Jiang H.; Xu D. Tunable Dual-Emission in Monodispersed Sb^3+^/Mn^2+^ Codoped Cs_2_NaInCl_6_ Perovskite Nanocrystals through an Energy Transfer Process. Small 2020, 16, 2002547 10.1002/smll.202002547. 32608156

[ref49] Oomen E. W. J. L.; Smit W. M. A.; Blasse G. On the Luminescence of Sb^3+^ in Cs_2_NaMCl_6_(with M = Sc,Y,La): A Model System for the Study of Trivalent s^2^ ions. J. Phys. C: Solid State Phys. 1986, 19, 3263–3272. 10.1088/0022-3719/19/17/020.

[ref50] Jing Y.; Liu Y.; Zhao J.; Xia Z. Sb^3+^ Doping-Induced Triplet Self-Trapped Excitons Emission in Lead-Free Cs_2_SnCl_6_ Nanocrystals. J. Phys. Chem. Lett. 2019, 10, 7439–7444. 10.1021/acs.jpclett.9b03035. 31726830

[ref51] Li J.; Tan Z.; Hu M.; Chen C.; Luo J.; Li S.; Gao L.; Xiao Z.; Niu G.; Tang J. Antimony Doped Cs_2_SnCl_6_ with Bright and Stable Emission. Front. Optoelectron. 2019, 12, 352–364. 10.1007/s12200-019-0907-4.

[ref52] Majher J. D.; Gray M. B.; Liu T.; Holzapfel N. P.; Woodward P. M. Rb_3_InCl_6_: A Monoclinic Double Perovskite Derivative with Bright Sb^3+^-Activated Photoluminescence. Inorg. Chem. 2020, 59, 14478–14485. 10.1021/acs.inorgchem.0c02248. 32960045

[ref53] Jing Y.; Liu Y.; Jiang X.; Molokeev M. S.; Lin Z.; Xia Z. Sb^3+^ Dopant and Halogen Substitution Triggered Highly Efficient and Tunable Emission in Lead-Free Metal Halide Single Crystals. Chem. Mater. 2020, 32, 5327–5334. 10.1021/acs.chemmater.0c01708.

[ref54] Han P.; Luo C.; Yang S.; Yang Y.; Deng W.; Han K. All-Inorganic Lead-Free 0D Perovskites by a Doping Strategy to Achieve a PLQY Boost from < 2% to 90%. Angew. Chem., Int. Ed. 2020, 59, 12709–12713. 10.1002/anie.202003234. 32337797

[ref55] Arfin H.; Kshirsagar A. S.; Kaur J.; Mondal B.; Xia Z.; Chakraborty S.; Nag A. ns^2^ Electron (Bi^3+^ and Sb^3+^) Doping in Lead-Free Metal Halide Perovskite Derivatives. Chem. Mater. 2020, 32, 10255–10267. 10.1021/acs.chemmater.0c03394.

[ref56] Liu X.; Xu X.; Li B.; Liang Y.; Li Q.; Jiang H.; Xu D. Antimony-Doping Induced Highly Efficient Warm-White Emission in Indium-Based Zero-Dimensional Perovskites. CCS Chem. 2020, 2, 216–224. 10.31635/ccschem.020.202000159.

[ref57] Pawley G. Unit-Cell Refinement from Powder Diffraction Scans. J. Appl. Crystallogr. 1981, 14, 357–361. 10.1107/S0021889881009618.

[ref58] Dean J. A.; Lange N. A. Lange’s Handbook of Chemistry; McGraw-Hill, 1999.

[ref59] Zhu D.; Zito J.; Pinchetti V.; Dang Z.; Olivati A.; Pasquale L.; Tang A.; Zaffalon M. L.; Meinardi F.; Infante I.; De Trizio L.; Manna L.; Brovelli S. Compositional Tuning of Carrier Dynamics in Cs_2_Na_1-x_Ag_x_BiCl_6_ Double-Perovskite Nanocrystals. ACS Energy Lett. 2020, 5, 1840–1847. 10.1021/acsenergylett.0c00914. 33344767 PMC7739488

[ref60] Meinardi F.; Bruni F.; Brovelli S. Luminescent Solar Concentrators for Building-Integrated Photovoltaics. Nat. Rev. Mater. 2017, 2, 17072 10.1038/natrevmats.2017.72.

