# Supplementary material for: Sb-Doped Metal Halide Nanocrystals: A 0D versus 3D Comparison
Source: ACS Energy Lett. 2021 May 27;6(6):2283–92. doi: 10.1021/acsenergylett.1c00789 (PMC8294020; doi:10.1021/acsenergylett.1c00789)
Supplement: Supplementary file 1 — nz1c00789_si_001.pdf [file nz1c00789_si_001.pdf]

SUPPORTING INFORMATION

Sb-Doped Metal Halide Nanocrystals: a 0D versus 3D comparison

Dongxu Zhu[a], Matteo L. Zaffalon[b], Juliette Zito[a,c], Francesca Cova[b], Francesco Meinardi[b], Luca De Trizio[a]*, Ivan Infante[a]*, Sergio Brovelli[b]*, Liberato Manna[a]*

[a] Nanochemistry Department Istituto Italiano di Tecnologia, Via Morego 30, 16163 Genova, Italy

[b] Dipartimento di Scienza dei Materiali, Università degli Studi di Milano Bicocca, Via R. Cozzi 55, 20125 Milano, Italy

[c] Dipartimento di Chimica e Chimica Industriale, Università degli Studi di Genova, 16146 Genova, Italy

EXPERIMENTAL SECTION

Chemicals. Cesium carbonate (Cs_2CO_3, 99%), rubidium carbonate (Rb_2CO_3, 99%), sodium acetate (Na(ac), 99%), potassium acetate (K(ac), 99%), indium(III) acetate($In(ac)_3$, 99.99%), antimony(III) acetate ($Sb(ac)_3$, 99.99%), oleylamine (OLAM, 98**%),** oleic acid (OA, 90%), dioctyl ether (DOE, 99%), benzoyl chloride (Bz-Cl, 98%), hexane (anhydrous 95%), ethyl acetate (99.8%) were purchased from Sigma-Aldrich. All chemicals were used without any further purification.

Synthesis of Sb-doped $Cs_2NaInCl_6$ NCs. In a typical synthesis, Cs_2CO_3(0.25 mmol), Na(ac)(0.25 mmol), $In(ac)_3$(0.25 mmol), 4 ml DOE, 1.2 ml OA and 0.6 ml OLAM were mixed in a 20 ml vial under N_2. Then, the system was heated on a hotplate up to 140 °C and at that temperature a solution of 220 µl Bz-Cl in 0.5 ml of degassed DOE was swiftly injected into the vial. Immediately after the injection, the reaction was cooled down by an ice-water bath. Then, 3 ml of hexane was added to the solution, which was then centrifuged at 4000 rpm for 5 min, and the supernatant was discarded. The precipitated NCs were redispersed in 3 mL of hexane, and centrifuged at 5500 rpm for 5 min. The precipitated NCs were discarded and the supernatant was precipitated by adding 3 ml ethyl acetate and centrifuged at 4000 rpm for 5 min. The precipitated NCs were dispersed in 1.5 ml of hexane and centrifuged at 5500 rpm for 5 min. The supernatant was stored in a vial for further characterizations. All the washing procedures were carried out under inert atmosphere. Sb-doped $Cs_2NaInCl_6$ NCs were obtained by the same protocol, but adding different amounts of $Sb(ac)_3$ while keeping all the reaction parameters fixed. The Sb/In feed ratio was varied from 0.5% to 1%, 5%, 10%, 20%.

Synthesis of Sb-doped Cs_2KInCl_6 NCs. The undoped and Sb-doped Cs_2KInCl_6 NCs were synthesized using the same method employed for the undoped and Sb-doped $Cs_2NaInCl_6$ NCs with minor modifications: Na(ac) was replaced by K(ac), 0.175 mmol of $In(ac)_3$ were used, and all the other reaction parameters were kept constant. The Sb/In feeding amount was varied from 0.25% to 1%, 5%, 10%, 20%.

Synthesis of Sb-doped Rb_3InCl_6 NCs. In a typical synthesis, Rb_2CO_3 (0.375 mmol), $In(ac)_3$((1-x)*0.25 mmol), $Sb(ac)_3$(x*0.25 mmol), 4 ml DOE, 1.2 ml OA, 0.6 ml OLAM were mixed in a in a 20 ml vial under N_2. Then, the system was heated on a hotplate up to 140 °C and at that temperature a solution of 220 µl Bz-Cl in 0.5 ml of degassed DOE was swiftly injected into the vial. Immediately after the injection, the reaction was cooled down by an ice-water bath. The washing and precipitation steps were similar to those employed for Sb doped $Cs_2Na(K)InCl_6$ NCs except for the

use of 6 ml (rather than 3 ml) of ethyl acetate. Here, the *x* values were selected as 0, 1%, 5%, 10%, 10% and 20%, respectively.

Transmission Electron Microscopy (TEM) analysis. The samples were prepared by dropping dilute NCs solutions on 200 mesh carbon-coated copper grids for low-resolution TEM. Low-resolution TEM measurements were performed on a JEOL JEM-1400Plus microscope with a thermionic gun (W filament) operated at an acceleration voltage of 120 kV.

X-ray Diffraction (XRD). XRD analysis was carried out on PANanalytical Empyrean X-ray diffractometer equipped with a 1.8 kW Cu Kα ceramic X-ray tube and a PIXcel^{3D} 2x2 area detector, operating at 45 kV and 40 mA. Specimens for the XRD measurements were prepared by dropping a concentrated NCs solution onto a silicon zero-diffraction single crystal substrate. The diffraction patterns were collected under ambient conditions using a parallel beam geometry and the symmetric reflection mode. Data analysis was carried out using PDXL 2.8.1.1 from Rigaku. The unit-cell parameters were refined by using the whole-powder-pattern decomposition (WPPD) technique based on the Pawley algorithm. All parameters were refined by the least-squares method. The pseudo-Voigt function was used as peak profile function.

Scanning Electron Microscopy (SEM). SEM analysis was performed on a HRSEM JEOL JSM-7500LA microscope with a cold field-emission gun (FEG), operating at 15 kV acceleration voltage. Energy-dispersive spectroscopy (EDS, Oxford instrument, X-Max, 80 mm^2) was used to evaluate the elemental ratios. All experiments were done at 8 mm working distance, 15 kV acceleration voltage and 15 sweep count for each sample.

Inductively Coupled Plasma (ICP-OES) Elemental Analysis. ICP elemental analysis, performed via inductively coupled plasma optical emission spectroscopy (ICP-OES) with an iCAP 6300 DUO ICP-OES spectrometer (ThermoScientific) was used to quantify the Sb to In ratio. All chemical analyses performed by ICP-OES were affected by a systematic error of about 5%. The samples were dissolved with 1 ml aqua regia (HCl/HNO_3=3/1(v/v)) overnight.

Optical Measurements. The absorption spectra were recorded using a Varian Cary 50 ultra violet-visible absorption spectrophotometer. The steady-state PL and PL excitation (PLE) spectra were measured on a Varian Cary Eclipse spectrophotometer. Time-resolved PL experiments were conducted exciting the samples with a frequency tripled pulsed Nd:YAG laser at 3.49 eV collecting with a Hamamatsu R943-02 time-correlated single-photon counting unit coupled to an Oriel Instruments Cornerstone 260 monochromator. The PL efficiencies were measured at room temperature comparing the PL intensity, under steady-state excitation at 320 nm, of an hexane dispersion of NCs, and the emission from Quinine Sulfate dissolved in 0.5 M H_2SO_4 used as standard reference material.[1]

Radio-Luminescence. 0.7% Sb-doped $Cs_2NaInCl_6$ NC, 0.9% Sb-doped Cs_2KInCl_6 NC and 0.8% Sb-doped Rb_3InCl_6 NC samples were excited by X-ray irradiation through a beryllium window, using a Philips 2274 X-ray diffraction tube (with a tungsten target) operated at 32 kV. The spectra were collected at room temperature with a homemade apparatus featuring a liquid nitrogen-cooled, back-illuminated and UV-enhanced, CCD detector (Jobin Yvon Symphony II) coupled to a monochromator (Jobin Yvon Triax 180) equipped with a 100 grooves/mm grating as detection system. The spectra were corrected for the spectral response of the acquisition system. The dose values reported in the text for X-ray irradiations were obtained by comparison with a calibrated 90Sr-90Y beta radioactive source and using optically stimulated luminescence emission from quartz crystalline powder (100-200 μm).

Computational Methodology. The electronic structure and absorption features of the Sb center were investigated using a simple $[Cs_8SbCl6]^{5+}$ model cleft from the optimized Sb-doped $Cs_2NaInCl_6$ bulk structure. Single point calculations at the DFT level were carried out using the PBE exchange−correlation functional[2] and double-ζ basis set extended with a polarization function (DZP) for all atoms,[3] as implemented in the ADF package.[4] Relativistic effects were taken into account by means of the zeroth-order regular approximated (ZORA) Hamiltonian.[5-6] Spin-orbit coupling was also included variationally from the outset. The first ten excited states were calculated by employing a time-dependent (TD-DFT) approach with the Tamm-Dancoff approximation (TDA)[7] and same functional and basis-set. The electronic structures of both DP ($Cs_2NaInCl_6$ and Cs_2KInCl_6) and 0D systems (Rb_3InCl_6) were modelled by preparing the corresponding 2x2x2 supercells and successively doping them by substituting one In^{3+} ion with an Sb^{3+} ion. The atomic positions and cell parameters were then relaxed at the gamma point at the DFT/PBE/DZVP[8] level of

theory using the CP2K 7.1 package.[9] Scalar relativistic effects were incorporated as effective core potentials, while spin-orbit coupling was neglected. Additionally, a systematic elongation of the axial Sb-Cl bonds from the ground state equilibrium value of 2.67 Å to a maximum value of 3.12 Å with steps of 0.025 Å was performed at the same level of theory.

Surface effects on DP systems were probed by modelling explicitly cubic shaped Sb-doped Cs_2AInCl_6 [A = Na, K] NCs of about 3.5 nm in side. The corresponding NCs models were created by cutting, respectively, the $Cs_2NaInCl_6$ and Cs_2KInCl_6 cubic bulk structures along the (100) directions, leaving Cs and Cl on the surface and ultimately obtaining a charge balanced $Cs_{324}A_{108}In_{108}Cl_{756}$ model. These NCs were then doped by introducing one Sb^{3+} ion (replacing one In^{3+} ion) in the core, leading to a final stoichiometry of $Cs_{324}A_{108}In_{107}SbCl_{756}$. As shown in Figure 4e, the size of these models is of about 3.5 nm, representing a good balance between their cost in terms of computational time and their pertinence from an experimental point of view. Structural relaxation and analysis of the electronic structure of the Sb-doped Cs_2AInCl_6 [A = Na, K] NCs were carried out again at the DFT/PBE/DZVP level of theory with CP2K 7.1 package.

To evaluate the equilibrium length of the axial Sb-Cl bonds in the excited state, we started from the relaxed Rb_3InCl_6 2x2x2 supercell doped with one Sb^{3+} ion and stretched the axial bonds of the Sb octahedron of about 0.6 Å. We then relaxed the atomic positions of the resulting supercell in the triplet state (a common computational strategy to mimic the lowest singlet excited state) at the DFT/PBE level of theory, finally obtaining an equilibrium length of about 3.05 Å for the axial Sb-Cl bonds.

Monte Carlo Ray-Tracing Simulation. The simulations of the waveguiding performances were performed via a Monte Carlo ray tracing method, in which the photon propagation follows the geometrical optics laws. Because the plastic scintillator thickness is much larger than the light coherence length, the interference has been neglected. The stochastic nature of the simulations is reflected in the fact that the ray is not split upon reaching an interface but it is either transmitted or reflected with the probabilities proportional to respective energy fluxes given by Fresnel Laws. The dependence of these probabilities on the state of polarization of the incident ray (e.g., s or p polarized) is also considered. Inside the scintillator material, for each ray, the inverse transform sampling method is applied to randomly generate the length of the optical path before absorption by the NCs. Path lengths follow the exponential attenuation law determined by the wavelength-dependent absorption cross section, $\sigma(\lambda)$, and the NC concentration ($N(\lambda)$,), via an attenuation coefficient, $k(\lambda)= k(\lambda)N(\lambda)$. Since the mean path length, given by the inverse attenuation coefficient, is always much greater than the average distance between NCs, there is no need to keep track of an explicit position of each NC, so the nanocomposite material (PMMA+ NCs) can be considered within the effective medium approach, i.e., as a uniform material with the attenuation coefficient defined above. Once a photon is absorbed by a NC, the subsequent fate of the excitation (i.e., reemission or nonradiative relaxation) is again determined by the Monte Carlo sampling according to the emission quantum yield. The direction of reemission is distributed uniformly and the reemission wavelength is determined using the rejection sampling applied to the accurate NC luminescence spectrum obtained from experiment.

The ultimate fate of each photon is either loss due to nonradiative recombination or escape from the scintillator via one of the interfaces. A single ray Monte Carlo simulation is typically repeated 10^5-10^7 times to have a proper statistical averaging. A stochastic nature of simulations allows one to easily evaluate various observables and add additional processes.

Table S1. Composition of Sb-doped $Cs_2NaInCl_6$ NCs measured by SEM-EDS and ICP-OES analyses. The stoichiometry of the samples was expressed in relation to the quantity of In, which was assumed to be 1.

Sb/In precursors ratio (%)	SEM-EDS composition	ICP-OES Sb/In (%)
0	$Cs_{2.09}Na_{0.94}InCl_{6.07}$	0
0.5	$Cs_{2.14}Na_{0.96}InSb_{0.01}Cl_{6.18}$	0.3
1	$Cs_{2.24}Na_{0.95}InSb_{0.01}Cl_{6.17}$	0.7
5	$Cs_{2.17}Na_{1.05}InSb_{0.04}Cl_{6.30}$	3.2
10	$Cs_{2.20}Na_{1.04}InSb_{0.05}Cl_{6.30}$	4.9
20	$Cs_{2.15}Na_{0.94}InSb_{0.08}Cl_{6.17}$	8.0

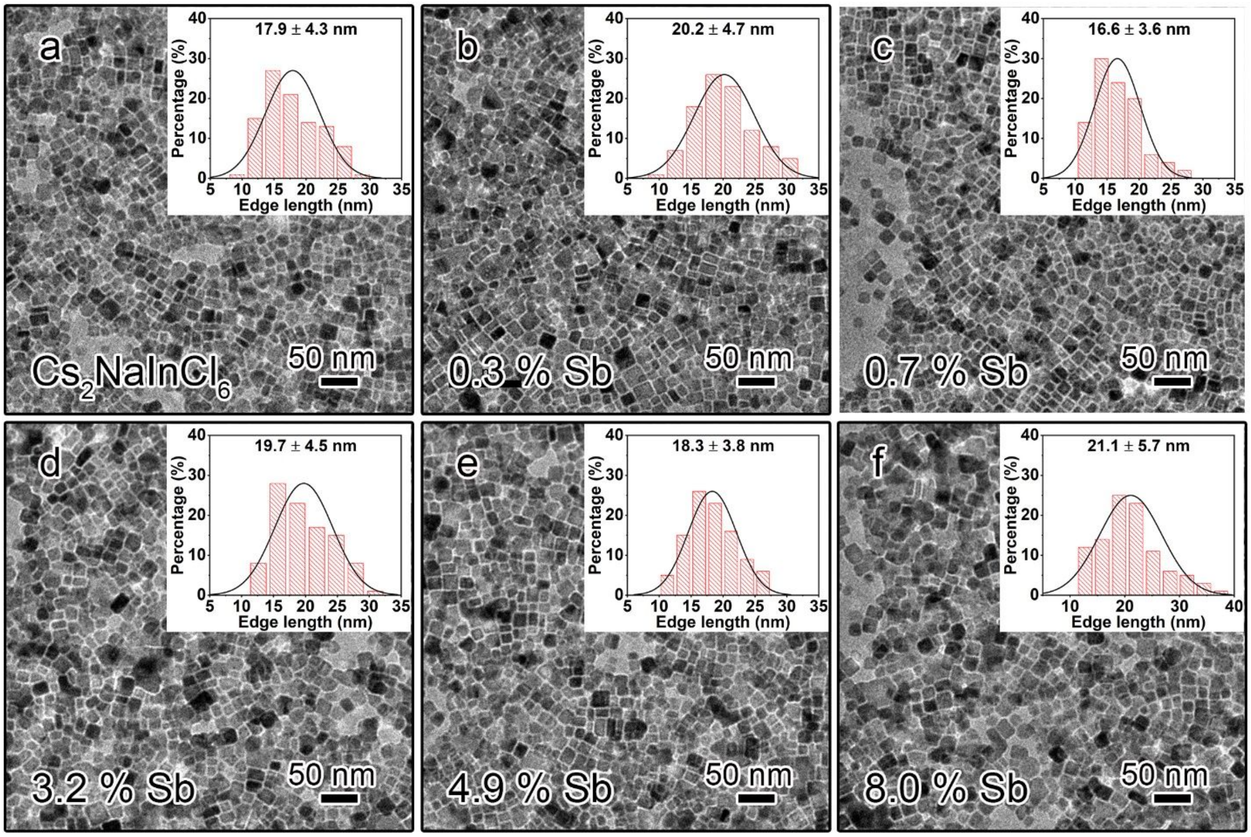

Figure S1. TEM images and corresponding size distribution histograms of (a) $Cs_2NaInCl_6$ NCs and Sb-doped $Cs_2NaInCl_6$ NCs with analytical Sb concentration of (b) 0.3 %, (c) 0.7 %, (d) 3.2 %, (e) 4.9 %, (f) 8.0 %. The Sb concentrations are defined as [Sb]/[In] and have been estimated by ICP-OES analysis.

Table S2. Composition of Sb-doped Cs_2KInCl_6 NCs measured by SEM-EDS and ICP-OES analyses. The stoichiometry of the samples was expressed in relation to the quantity of In, which was assumed to be 1.

Sb/In precursors ratio (%)	SEM-EDS composition	ICP-OES Sb/In (%)
0	$Cs_{2.39}K_{0.92}InCl_{6.73}$	0
0.25	$Cs_{2.33}K_{0.84}InCl_{6.49}$	0.1
1	$Cs_{2.40}K_{0.92}InCl_{6.71}$	0.9
5	$Cs_{2.20}K_{0.99}InCl_{6.63}$	1.6
10	$Cs_{2.31}K_{0.95}InSb_{0.01}Cl_{6.73}$	3.3
20	$Cs_{2.34}K_{0.99}InSb_{0.05}Cl_{6.74}$	6.3

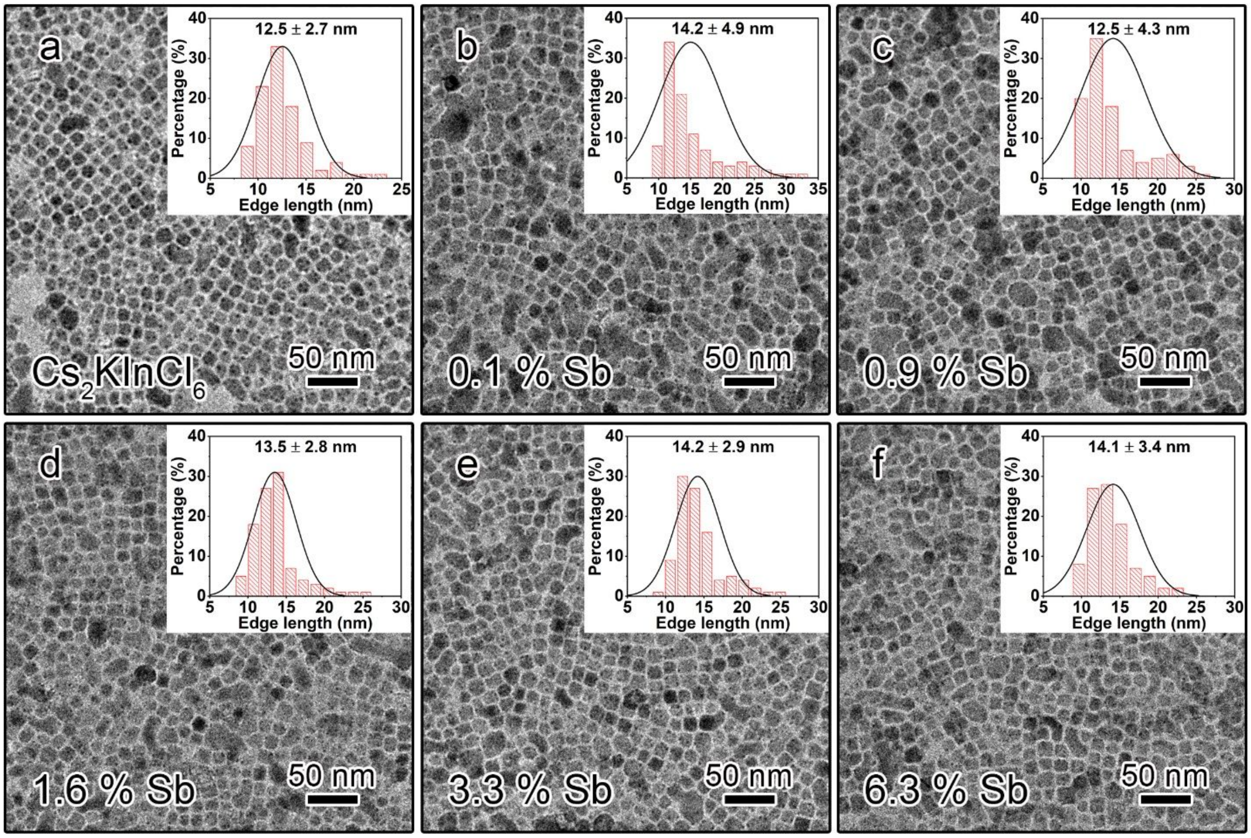

Figure S2. TEM images and corresponding size distribution histograms of (a) Cs_2KInCl_6 NCs and Sb-doped Cs_2KInCl_6 NCs with analytical Sb concentration of (b) 0.1 %, (c) 0.9 %, (d) 1.6 %, (e) 3.3 %, (f) 6.3 %. The Sb concentrations are defined as [Sb]/[In] and have been estimated by ICP-OES analysis.

Whole-Powder-Pattern Decomposition (WPPD)

The unit-cell parameters were refined by using the WPPD technique based on the Pawley algorithm. All parameters were refined by the least-squares method. The pseudo-Voigt function was used as peak profile function.

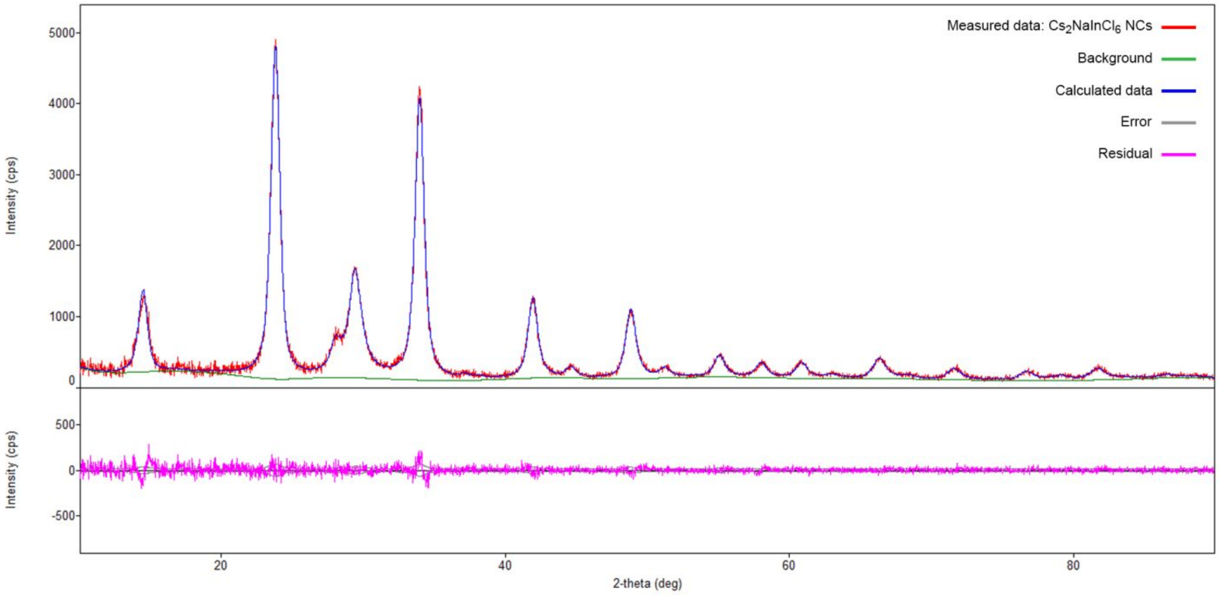

**a**	10.533(5) Å
**b**	10.533(5) Å
**c**	10.533(5) Å
**alpha**	90°
**beta**	90°
**gamma**	90°
**Volume**	1168.6(9) $Å^3$
**S**	1.9933

Figure S3. Refinement results of $Cs_2NaInCl_6$ NCs using the WPPD method.

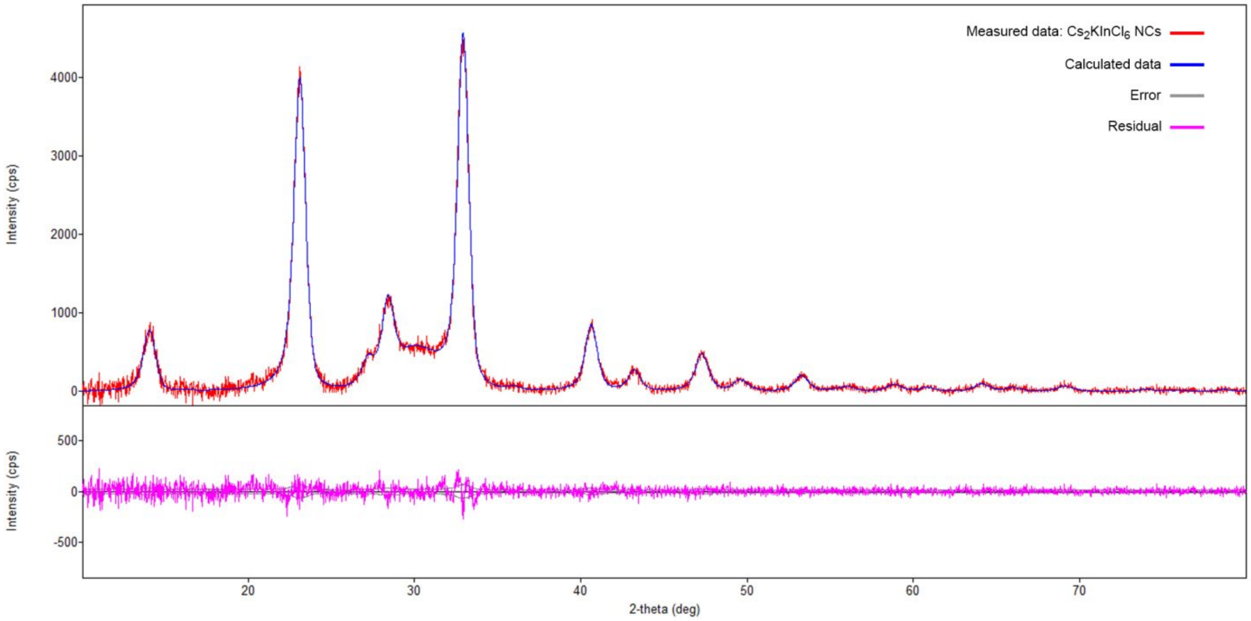

**a**	10.871(4) Å
**b**	10.871(4) Å
**c**	10.871(4) Å
**alpha**	90°
**beta**	90°
**gamma**	90°
**Volume**	1284.7(7) Å^3
**S**	2.2524

Figure S4. Refinement results of Cs_2KInCl_6 NCs using the WPPD method.

Table S3. Composition of Sb-doped Rb_3InCl_6 NCs measured by SEM-EDS and ICP-OES analyses. The stoichiometry of the samples was expressed in relation to the quantity of In, which was assumed to be 1.

Sb/In precursor ratio (%)	SEM-EDS composition	ICP-OES Sb/In (%)
0	$Rb_{2.49}InCl_{5.92}$	0
1	$Rb_{2.37}InCl_{5.96}$	0.1
5	$Rb_{2.47}InCl_{6.16}$	0.8
10	$Rb_{2.31}InCl_{6.16}$	1.1
15	$Rb_{2.44}InCl_{6.05}$	1.7
20	$Rb_{2.41}InCl_{6.12}$	3.0

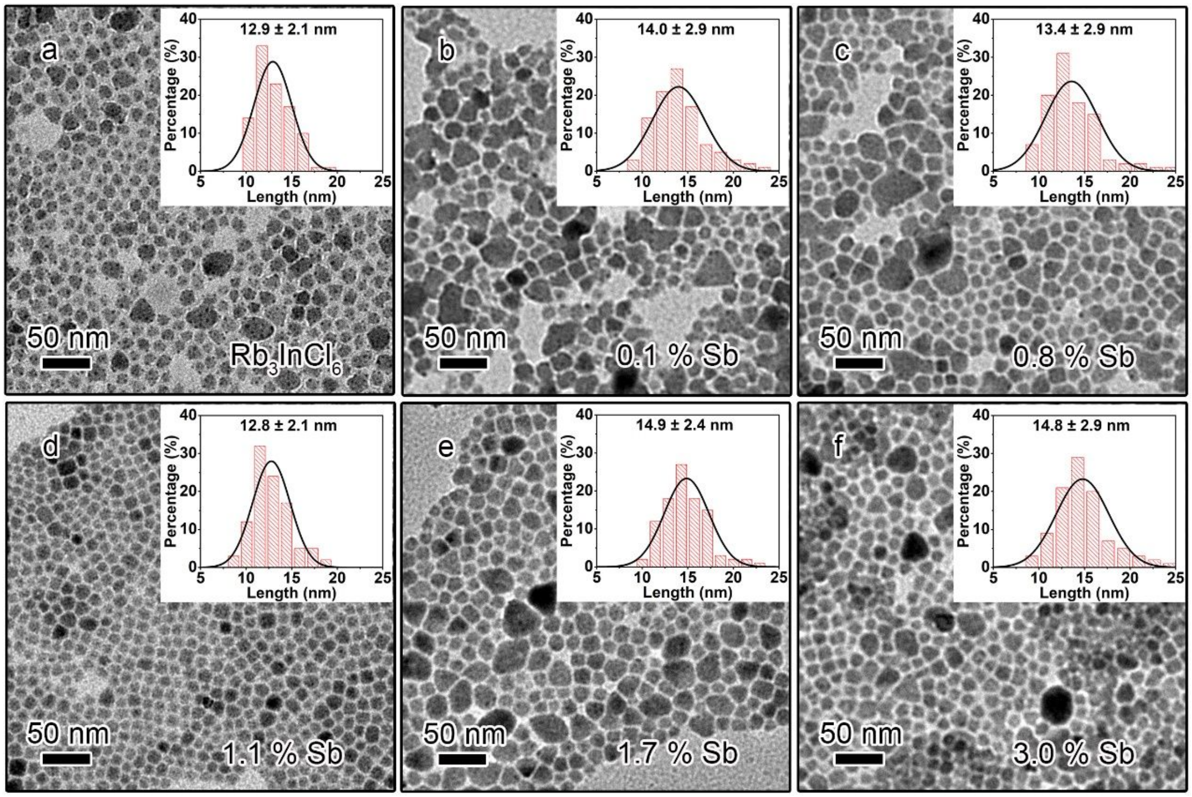

Figure S5. TEM images and corresponding size distribution histograms of (a) Rb_3InCl_6 NCs and Sb-doped Rb_3InCl_6 NCs with analytical Sb concentration of (b) 0.1 %, (c) 0.8 %, (d) 1.1 %, (e) 1.7 %, (f) 3.0 %. The Sb concentrations are defined as [Sb]/[In] and have been estimated by ICP-OES analysis.

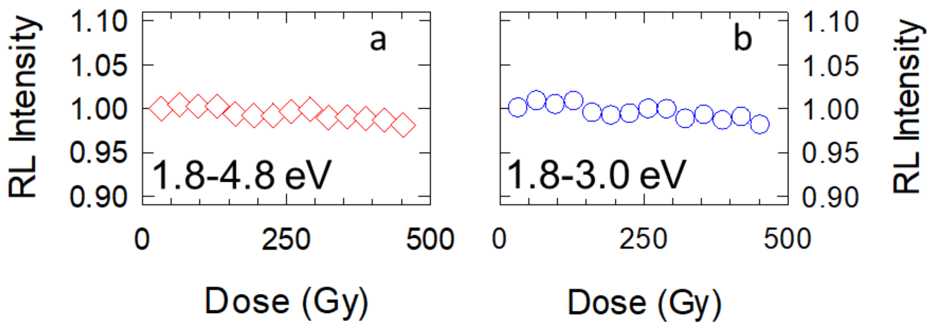

Figure S6. Integrated RL intensity of 0.8% Sb-doped Rb_3InCl_6 NCs at increasing cumulated dose up to 500 Gy, with a dose rate of 0.5 Gy/s. Data are normalized to the initial value and each data point represents the integral of the RL emission spectrum in the (a) 1.8 – 3.0 eV or (b) 1.8-30 eV range.

References

(1) Eaton, D. F., Reference Materials for Fluorescence Measurement. *Pure Appl. Chem.* **1988,** *60*, 1107-1114.

(2) Perdew, J. P.; Burke, K.; Ernzerhof, M., Generalized Gradient Approximation Made Simple. *Phys. Rev. Lett.* **1996,** *77*, 3865-3868.

(3) Van Lenthe, E.; Baerends, E. J., Optimized Slater-Type Basis Sets for the Elements 1–118. *J. Comput. Chem.* **2003,** *24*, 1142-1156.

(4) te Velde, G.; Bickelhaupt, F. M.; Baerends, E. J.; Fonseca Guerra, C.; van Gisbergen, S. J. A.; Snijders, J. G.; Ziegler, T., Chemistry with Adf. *J. Comput. Chem.* **2001,** *22*, 931-967.

(5) Wang, F.; Ziegler, T.; Lenthe, E. v.; Gisbergen, S. v.; Baerends, E. J., The Calculation of Excitation Energies Based on the Relativistic Two-Component Zeroth-Order Regular Approximation and Time-Dependent Density-Functional with Full Use of Symmetry. *J. Chem. Phys.* **2005,** *122*, 204103.

(6) Wang, F.; Ziegler, T., A Simplified Relativistic Time-Dependent Density-Functional Theory Formalism for the Calculations of Excitation Energies Including Spin-Orbit Coupling Effect. *J. Chem. Phys.* **2005,** *123*, 154102.

(7) Hirata, S.; Head-Gordon, M., Time-Dependent Density Functional Theory within the Tamm–Dancoff Approximation. *Chem. Phys. Lett.* **1999,** *314*, 291-299.

(8) VandeVondele, J.; Hutter, J., Gaussian Basis Sets for Accurate Calculations on Molecular Systems in Gas and Condensed Phases. *J. Chem. Phys.* **2007,** *127*, 114105.

(9) Kühne, T. D.; Iannuzzi, M.; Ben, M. D.; Rybkin, V. V.; Seewald, P.; Stein, F.; Laino, T.; Khaliullin, R. Z.; Schütt, O.; Schiffmann, F.; Golze, D.; Wilhelm, J.; Chulkov, S.; Bani-Hashemian, M. H.; Weber, V.; Borštnik, U.; Taillefumier, M.; Jakobovits, A. S.; Lazzaro, A.; Pabst, H.; Müller, T.; Schade, R.; Guidon, M.; Andermatt, S.; Holmberg, N.; Schenter, G. K.; Hehn, A.; Bussy, A.; Belleflamme, F.; Tabacchi, G.; Glöß, A.; Lass, M.; Bethune, I.; Mundy, C. J.; Plessl, C.; Watkins, M.; VandeVondele, J.; Krack, M.; Hutter, J., Cp2k: An Electronic Structure and Molecular Dynamics Software Package - Quickstep: Efficient and Accurate Electronic Structure Calculations. *J. Chem. Phys.* **2020,** *152*, 194103.
